# Comparison of software tools for kinetic evaluation of chemical degradation data

**DOI:** 10.1186/s12302-018-0145-1

**Published:** 2018-05-18

**Authors:** Johannes Ranke, Janina Wöltjen, Stefan Meinecke

**Affiliations:** 1 Kronacher Str. 12, Grenzach-Wyhlen, Germany; 2 German Environment Agency (UBA), Wörlitzer Platz 1, 06844 Dessau-Roßlau, Germany

**Keywords:** Kinetic evaluation, Software, Chemical degradation, Biodegradation

## Abstract

**Background:**

For evaluating the fate of xenobiotics in the environment, a variety of degradation or environmental metabolism experiments are routinely conducted. The data generated in such experiments are evaluated by optimizing the parameters of kinetic models in a way that the model simulation fits the data. No comparison of the main software tools currently in use has been published to date. This article shows a comparison of numerical results as well as an overall, somewhat subjective comparison based on a scoring system using a set of criteria. The scoring was separately performed for two types of uses. Uses of type I are routine evaluations involving standard kinetic models and up to three metabolites in a single compartment. Evaluations involving non-standard model components, more than three metabolites or more than a single compartment belong to use type II. For use type I, usability is most important, while the flexibility of the model definition is most important for use type II.

**Results:**

Test datasets were assembled that can be used to compare the numerical results for different software tools. These datasets can also be used to ensure that no unintended or erroneous behaviour is introduced in newer versions. In the comparison of numerical results, good agreement between the parameter estimates was observed for datasets with up to three metabolites. For the now unmaintained reference software DegKinManager/ModelMaker, and for OpenModel which is still under development, user options were identified that should be taken care of in order to obtain results that are as reliable as possible. Based on the scoring system mentioned above, the software tools gmkin, KinGUII and CAKE received the best scores for use type I. Out of the 15 software packages compared with respect to use type II, again gmkin and KinGUII were the first two, followed by the script based tool mkin, which is the technical basis for gmkin, and by OpenModel.

**Conclusions:**

Based on the evaluation using the system of criteria mentioned above and the comparison of numerical results for the suite of test datasets, the software tools gmkin, KinGUII and CAKE are recommended for use type I, and gmkin and KinGUII for use type II. For users that prefer to work with scripts instead of graphical user interfaces, mkin is recommended. For future software evaluations, it is recommended to include a measure for the total time that a typical user needs for a kinetic evaluation into the scoring scheme. It is the hope of the authors that the publication of test data, source code and overall rankings foster the evolution of useful and reliable software in the field.

**Electronic supplementary material:**

The online version of this article (10.1186/s12302-018-0145-1) contains supplementary material, which is available to authorized users.

## Background

The relevance of the degradation of chemicals in the environment for the evaluation of their risks for human health and the environment has been recognized more than 40 years ago [1]. The active ingredients of plant protection products were among the first anthropogenic chemicals for which a regulatory requirement was established to investigate not only the degradation of the parent compound, but also the formation and decline of their transformation products (TPs) in the environment. Note that TPs of pesticides and biocides are often termed metabolites, while for pharmaceuticals, the term metabolites is reserved for metabolites formed in the human body.

**Fig. 1:**
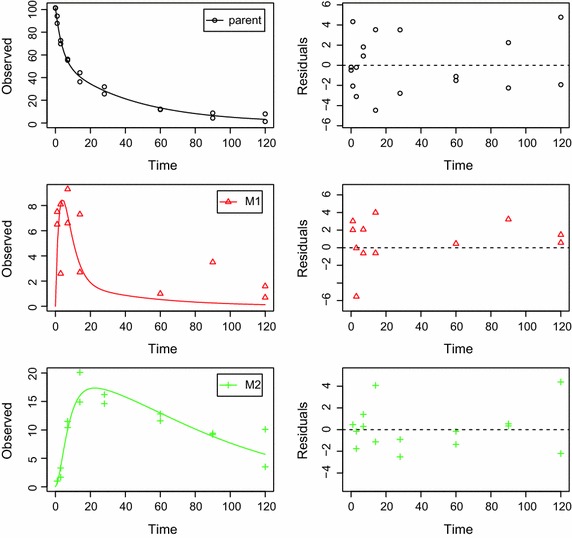
Example plot of a fitted model with two transformation products The observed and fitted time course of the concentration of the different chemical compounds is shown in the left panels. The deviations between observed and fitted curve are shown in the right panels

In 2006, a group of scientist from academia, governmental authorities and industry published a guidance document containing detailed recommendations on how the degradation in the corresponding environmental metabolism experiments should be evaluated [2]. For the sake of brevity, this guidance in its last revision from 2014 [3] is termed FOCUS guidance in the following. Briefly, such an evaluation according to FOCUS guidance consists of the definition of one or more mathematical models and the search of model parameters for these models resulting in model simulations with minimized deviations between simulated and observed data. This deviation is expressed as the sum of all squared differences between simulated and observed values (sum of squared residuals). While equal weight is given to all residuals in the first step, different weights can then be attributed to the residuals, based on the magnitude of the observed concentration or the chemical identity of the compound observed. A graphical representation of an unweighted fit for a dataset on a parent compound with two TPs is shown in Fig. 1.

Since the first publication of this guidance, a number of computer programs have been developed that facilitate the kinetic evaluation of chemical degradation data, using the model nomenclature established in the guidance, and providing not only numeric endpoints for the fitted degradation models like DT$_{50}$ and DT$_{90}$ values, but also statistical criteria to judge the uncertainty in the model parameters, like the *p* value of a *t* test for significant difference from zero, and goodness-of-fit measures.

Some of the software tools commonly used in the beginning are not maintained anymore, and a number of new packages have been developed. Some of these newer tools are already widely used by industry and governmental authorities. However, no comparison of their numerical accuracy or usability has been published to date. Therefore, the German Federal Environment Agency (UBA) has commissioned a comparative evaluation of the tools that are currently being actively maintained. This evaluation included a search for available software tools, a pre-screening step to narrow down the list of candidates based on exclusion criteria, a numerical software check based on the kinetic evaluation of a number of example datasets, and a final quantitative ranking based on a system of weighted scores that was established within the project.

The scoring was separately performed for two types of uses. Uses of type I are routine evaluations involving standard kinetic models and up to three metabolites in a single compartment. Evaluations involving non-standard model components, more than three metabolites or more than a single compartment belong to use type II. For use type I, usability is most important, while the flexibility of the model definition is most important for use type II.

## Methods

In this section, the evaluation criteria, the software packages and the example datasets are described that were used in the comparative evaluation.

### Exclusion criteria for the screening step

For a first screening of the software packages, 16 exclusion criteria were defined describing conditions that the packages had to fulfil in order to be eligible for the further evaluation.

Many of them were satisfied by all candidates. For example, all software packages were available for the Windows operating system, all were able to import and export data, and in all packages it was possible at least in principle to fit the degradation models to the data, to report the results and to calculate derived quantities.

However, out of these 16 exclusion criteria, a few actually led to the exclusion of specific software packages. These criteria are listed in Table 1. Notably, the requirement for use type I to support the fitting process with a graphical user interface (GUI) led to the exclusion of a large number of packages for this use type.

**Table 1:** Relevant exclusion criteria used for screening the software packages

Criterion	Use type I	Use type II	Comment
Software availability	Required	Required	Some packages could not be obtained
Software maintenance	Required	Required	Some packages were unmaintained
Models with ≥ 3 transformation products	Required	Required	Some packages had a restricted scheme
Use of model templates	Required	Required	Support for reuse of models
Graphical user interface (GUI)	Required		GUI with support for the fitting process
Support for complex models		Required	Support for backtransfer (multi-compartment models)

### Criteria for the scoring system

For the quantitative comparison of the software packages, a scoring system was developed. In this system, various beneficial attributes that a software could either have or not have are grouped under different topics. These attributes were collected and their importance was discussed during two meetings of degradation experts within the German Environment Agency. The exact numerical score connected with each attribute was then decided by the authors in a somewhat subjective procedure. The scores connected with the attributes were different for use types I and II. All criteria and the scores attributed to them are shown in Table 2.

**Table 2:** Criteria, attributes and their scores used for the quantitative comparison

Criterion	Attribute	Score I	Score II
General information			
Licence cost for 3 years	No cost	10	10
	40 users < 10,000 EUR	10	
	40 users < 30,000 EUR	5	
	10 users < 10,000 EUR		10
	10 users < 30,000 EUR		5
Source code availability	Calculations Open Source	15	15
	GUI Open Source	5	5
Operating system	Cross-platform	5	5
Add-ins	Available		2
Programmability	Available	5	5
Functionality and performance			
Number of transformation products that can be modelled	≥ 4	8	4
	≥ 10	2	6
	Unlimited		10
Weighting of data points	Possible to implement	5	5
	Implemented	5	5
Identification of outliers	Implemented	2	2
Kinetic models	SFO, FOMC, HS, DFOP	25	10
	Monod kinetics	2	2
Kinetic endpoints	DT50/DT90, formation fractions	25	10
Statistical endpoints	$\chi ^2$ error level	10	5
	Confidence intervals	8	5
	*t* test	10	5
Further statistics	Likelihood ratio test	2	2
	Log transformed rate constants	2	2
	DT*x* with free *x*	1	1
Kinetic sorption	Possible to implement		5
Complex models	Forcing data like temperature or moisture		10
	Several compartments possible	8	$^a$
	Repeated dosing		10
Optimisation algorithms	More than one choice		10
	MCMC possible to add		10
	MCMC implemented	0	10
Iteratively reweighted least squares	IRLS implemented	5	10
Time needed for optimisations	Possibility to abort	5	8
	Graphical progress display	5	5
Stability	No crashes	5	8
	Useful error messages	5	5
User interface and usability			
Grafical user interface (GUI)	Available	$^a$	15
	Clickable degradation pathways	10	5
	Selection of submodels	5	5
Fixing parameters	Implemented	5	5
	Easy to use	5	5
Reusable model templates	Storable	5	10
	Easy to use	5	5
Data import	Possible	5	5
	Copy and paste from MS Excel$^{TM}$	5	10
Documentation and Help			
Output	Regulatory information	10	5
	Information for reproducibility	5	5
Graphics	Save in EMF file format	2	2
	Zoom with mouse	3	3
Manual or online help	Available	5	8
	Well structured	5	10
Tutorial	Available	2	2
	Well structured	3	3
Training by supplier	Kinetics training	5	5
Maintenance and development	Contact person	0	5
	Bug processing	4	4
	Public version control system	2	2
Sum of scores		286	341

$^a$This was an exclusion criterion for this use type

*GUI* graphical user interface; SFO, FOMC, HS, DFOP: kinetic models [2]; DT_50_/DT_90_: time to degrade to 50/90%; DT*x*: Time to degrade to *x*%; *MCMC* Markov Chain Monte Carlo method [4]; *IRLS* iteratively reweighted least squares [5]; *MS* Microsoft Corporation; *EMF* enhanced metafile graphics file format

The decision if a criterion was fulfilled or not was agreed between two of the authors of this manuscript and later reviewed by the third author.

For each software package, scores for the respective use types I and II were summed up separately and expressed as percentages of the possible maximum scores. All scores and calculations are available as a spreadsheet file in Additional file 2.

### Software packages

**Fig. 2:**
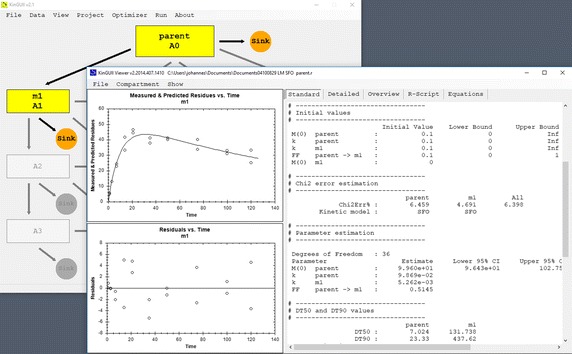
Screenshot of KinGUII v2.1 The model definition is shown on the left and the result viewer with a graph and some numerical results on the right

**Fig. 3:**
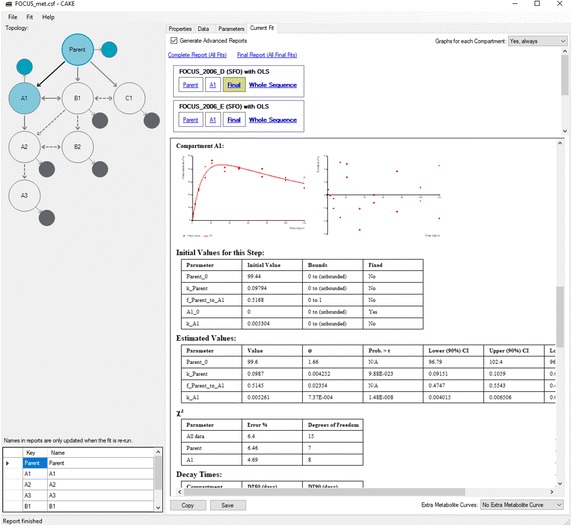
Screenshot of CAKE 3.3 The model definition is shown on the left and part of the summary report with a plot and some numerical results on the right

**Fig. 4:**
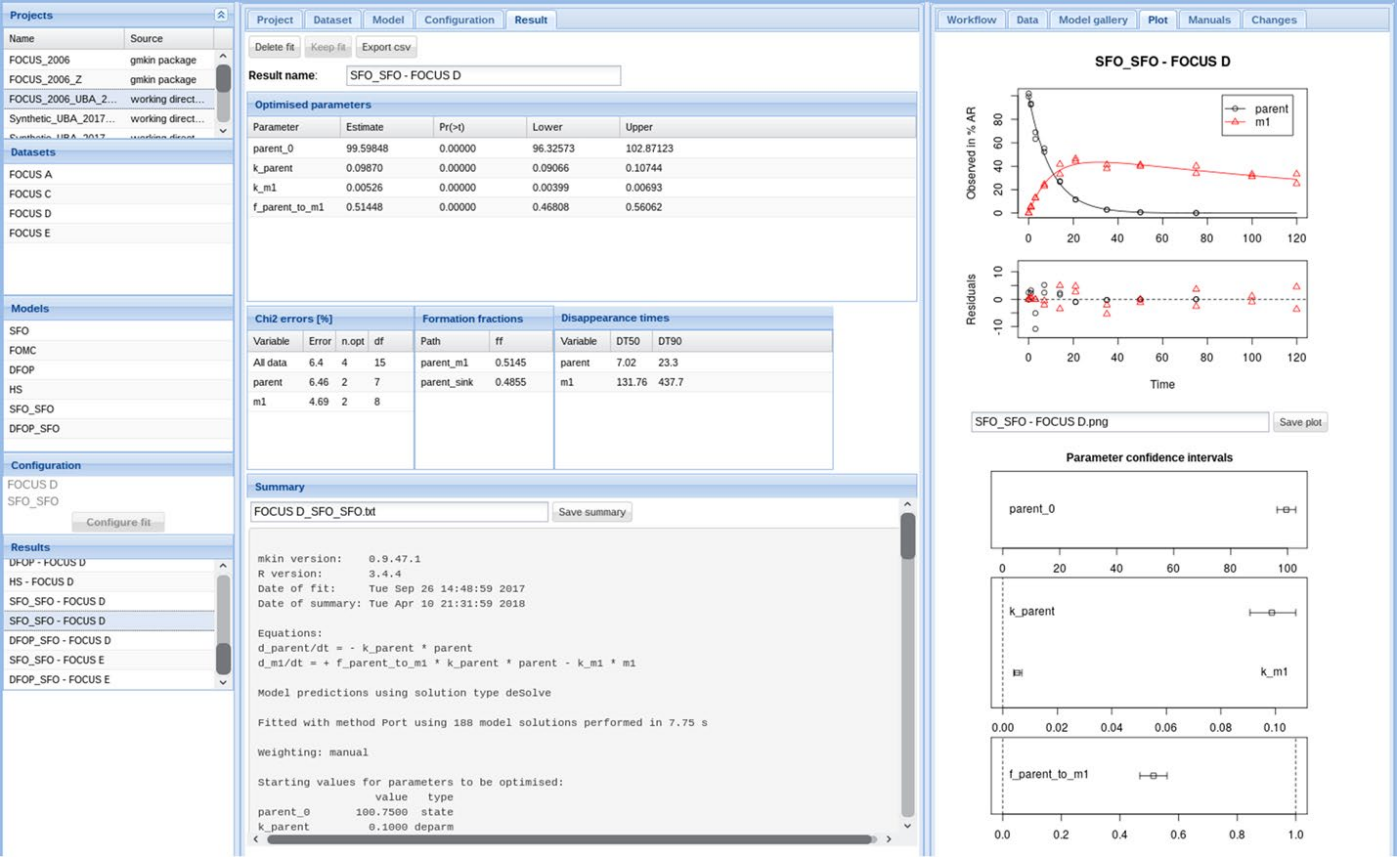
Screenshot of gmkin 0.6.7 Datasets and models are listed on the left, numerical results and a summary are shown in the center and a plot of the fitted model is on the right

**Fig. 5:**
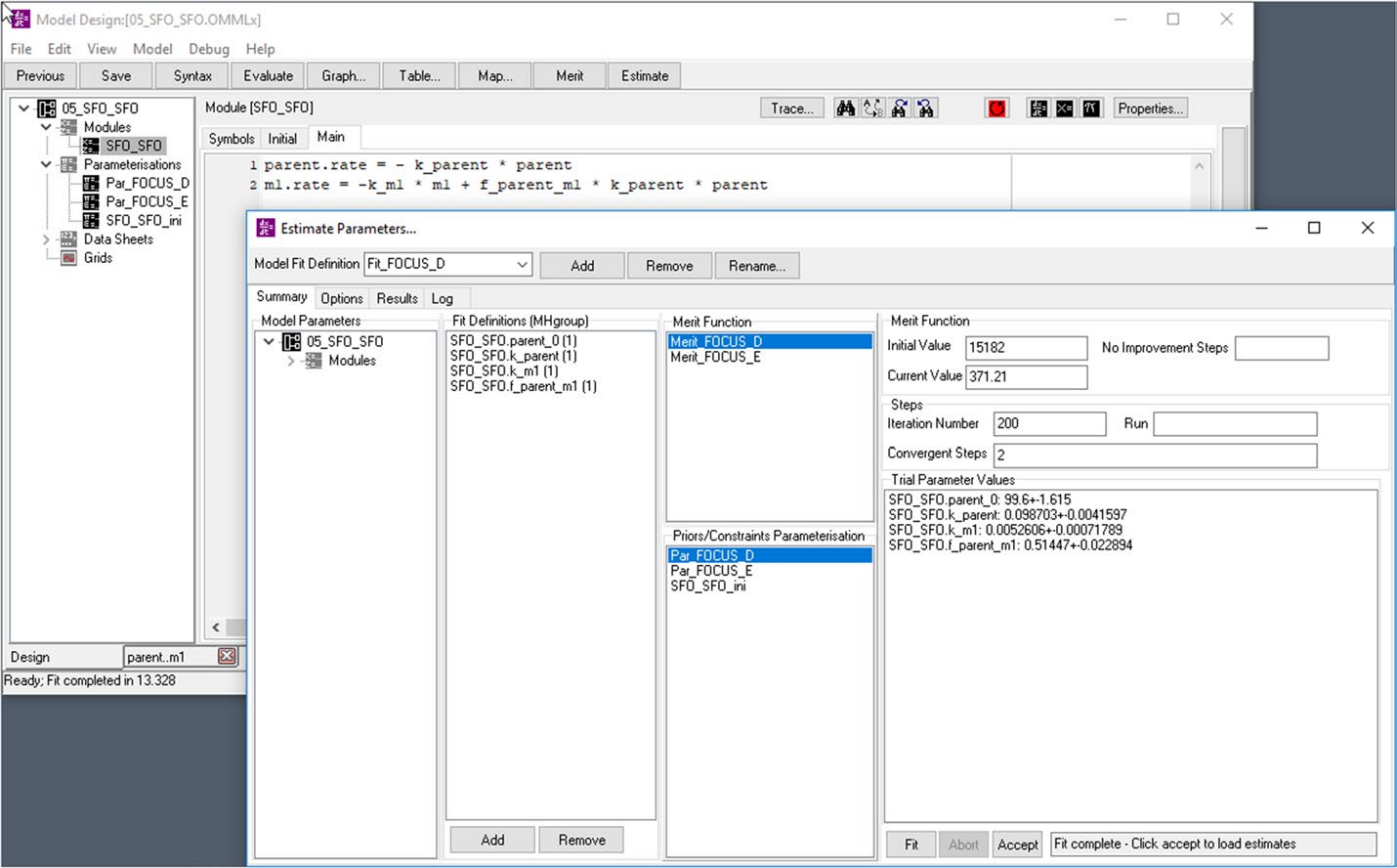
Screenshot of OpenModel 2.4.2 The script based model definition is shown in the main window on the left and the parameter estimation window is shown on the right

The software packages that were evaluated in-depth after the preselection step are described in the following. KinGUII v2.1 [6] (Fig. 2), available from Bayer AG, is internally using the R software [7] in version 3.0.1. The code used by KinGUII to do the actual fitting is written in R and published under an Open Source licence, as it was derived from one of the the first published versions of the mkin package [8] which is currently at version 0.9.47.1. CAKE 3.2 [9] (Fig. 3), available from Tessella Limited, is internally using R in version 3.0.1. The R code used by CAKE is also published under an Open Source licence, as it is based on a previous version of the R code used by KinGUII. The R package gmkin 0.6.8 [10] (Fig. 4) as published at the first author’s own website internally uses the latest version of mkin available at the Comprehensive R Archive Network (CRAN) at the time of installation, or at the latest R package update. OpenModel 2.4.2 [11] (Fig. 5) is available from the website of the University of Nottingham. While KinGUII, CAKE and gmkin have a common origin in the mkin codebase and are all employing the R software, the codebase of the three tools has diverged in a number of aspects like model formulation and optimization algorithms, so they have to be evaluated as independent tools.

In additon, the following software packages were evaluated for use type II (in no particular order): Origin and OriginPro, Maple and MapleSim, MATLAB with the Statistics Toolbox, or with the Statistics and the Optimizer Toolbox, Mathematica, SystemModeler, SciPy, SciLab and the R extension packages FME and mkin.

For the comparison of numerical results, also the software package DegKin Manager in version 1.0 from 28 September 2011 was included, together with ModelMaker 4.0.0, published in 2000, which is used by DegKin Manager for the actual integration and optimisation. These programs served as a reference, as they were previously used at the UBA.

### Test data

Four of the datasets that were used in the comparison of numerical results were taken from the FOCUS guidance [2], and 12 were synthetically generated in the course of the project as described below. In addition, two datasets from studies of the transformation in aquatic sediment systems (water sediment studies) and one dataset from an aerobic soil metabolism study were provided by UBA.

While it is widely accepted that separate degradation parameters for the water and the sediment phase in water sediment studies can not precisely be determined using the data generally available from such studies [12], such evaluations are still performed because they are requested by the FOCUS guidance. All datasets have been published as part of the mkin package [8].

The 12 new synthetic datasets were generated using four different kinetic models combined with three different error models. Regarding the kinetic models, the parent compound followed either simple first-order (SFO) or double first-order in parallel (DFOP, also called biexponential) kinetics, and was transformed either to two metabolites formed in sequential order, or to two metabolites formed in parallel. A graphical representation of these four kinetic models is shown in Additional file 1.

To each of them, three different stochastic components were added. In variants A and B, a normally distributed error component with a constant standard deviation $\sigma$ of magnitude of 3 and 7 was added, respectively. In relation to the starting concentration of 100, this is equivalent to coefficients of variation of 3 and 7%, respectively. The error model used in the generation of these datasets is therefore compatible with the assumptions used when using ordinary nonlinear least squares regression without weighting (termed ’OLS’ in CAKE, and ’NLLS’ in KinGUII, no weighting in gmkin).

In variant C, a heteroscedastic component was added, according to the two-component error model for analytical measurements of Rocke and Lorenzato [13]. This error model also assumes normal distribution of errors, but with a standard deviation $\sigma (y)$ that depends on the magnitude of the measured value *y* and has two components. The first component is the standard deviation at low measured values $\sigma _{\text {low}}$ and is independent of the measured value. The second component converges to an approximately constant relative standard deviation at high measured values $\mathrm {rsd}_{\text {high}}$, i.e. the increase in the total standard deviation is approximately proportional to the increase in the measured value.

$$\begin{aligned} \sigma (y) = \sqrt{\sigma _{\text {low}}^2 + y^2 \ \mathrm {rsd}_{\text {high}}^2} \end{aligned}$$

For the generation of the synthetic datasets in variant C using this error model, $\sigma _{\text {low}}$ was chosen to be 0.05, and $\mathrm {rsd}_{\text {high}}$ was chosen to be 0.07. The standard deviation for the time zero values with an absolute mean value of 100 according to this model is 7.0002, and therefore very similar to the standard deviation used for variant B. The standard deviation for an absolute mean value of 1 according to this error model is 0.086.

When the combination of the kinetic model predictions and the stochastic error model resulted in values below an assumed limit of detection of 0.1, these values were treated as not available (NA). A slightly generalised version of the R code used to generate them has been published as part of the mkin package in the documentation of the datasets [14]. Here, also the parameters used for the deterministic part of the model are listed.

The use of such synthetic datasets has the advantage that it can be checked if the parameters used to generate the data (deterministic part of the model) are found.

### Comparison of numerical results obtained for the test suite

In the comparison of numerical results using the datasets described above, some software settings were adjusted. For ModelMaker (used by DegKin Manager) and OpenModel, the stop value for the output time was set to the last sampling time in the respective test dataset. This was not necessary for the other software packages.

For the integration of kinetic models with ModelMaker, Runge-Kutta integration with 200 output points was used, the integration accuracy was set to 0.001 and constant error scaling was specified. For the automatic steplength calculation with OpenModel, an error factor of at least 1e−5 was specified.

For the optimisation, the settings that were predefined in the model files supplied by DegKin manager were generally not changed. For the termination criterion, the value for the fractional change was 0.01 in some model files and 0.001 in others. In OpenModel, the change threshold for the convergence was set to 1e−5 and the maximum number of iterations was set to 200.

No weighting methods were enabled. All evaluations were performed by the same person. Care was taken to avoid mistakes, but input or transcription errors can not completely be excluded.

## Results

In order to select the packages that were tested for numerical accuracy of the fitting, the quantitative criteria were applied to the software versions available. For use type I, the packages KinGUII, gmkin, CAKE and OpenModel were the only eligible packages as they fulfilled the corresponding requirements. Additional packages considered eligible for use type II are listed above in "Methods" section. From the list of packages eligible for use type II, the top five packages were KinGUII, gmkin, mkin, FME and OpenModel. The exact scores for all packages are available in a spreadsheet file as Additional file 2. As the R packages mkin and FME [15] are used internally by gmkin, these did not have to be evaluated separately in the comparison of numerical results.

### Comparison of numerical results

For the comparison of numerical results obtained for the test datasets, the tools that had the best scores for both use types were selected. These were CAKE, gmkin, KinGUII and OpenModel (in alphabetical order). As noted above, the in-house solution previously used at UBA, consisting of the tool DegKin Manager and the underlying ModelMaker Software, was inclued as a reference. However, the results obtained with the latter often deviated slightly, presumably because the optimisation settings were not adapted. In the following, mainly the results for the other four tools are discussed, because ModelMaker is not actively developed anymore.

Numerical results for the test datasets described above are shown in Additional file 1. Note that for mkin, these results have been obtained without using the graphical user interface gmkin for practical reasons.

For OpenModel, a number of settings had to be adapted in order to obtain reliable results. These are described above in the Methods section.

In the comparison of the results obtained for simple parent decline kinetics without metabolites applying the SFO, first-order multi-compartment (FOMC), DFOP and hockey stick (HS) models as defined in the FOCUS guidance, the following observations were made.

When the SFO model was fitted, the parameters obtained with KinGUII, CAKE, gmkin and OpenModel matched perfectly up to three significant digits. Only the parameters obtained with DegKin Manager deviated slightly, presumably due to suboptimal integration accuracy and convergence tolerance settings.

For the FOMC model, different observations were made, depending in the type of data that were evaluated. In cases where the degradation gets slower towards later sampling times, which is what the FOMC model was designed to address [16], the parameters obtained with CAKE, gmkin, KinGUII and OpenModel again matched exactly up to three significant digits. In contrast, in cases where the data were well described by simple first-order kinetics, the FOMC with its three parameters was ill-defined and the values of parameters $\alpha$ and $\beta$ varied widely across software tools, while their ratio was approximately the same for the different tools. Such cases of overparameterisation were already documented in similar comparisons involving the kinfit package [17].

Due to the strong correlation of parameters $\alpha$ and $\beta$ in the described cases of overparameterisation, as visible in the correlation matrix that is returned by the tools, the confidence intervals generated by the packages also widely differ. It may be worth to note that in one case (synthetic dataset SFO_lin_c), KinGUII reported confidence intervals for $\alpha$ and $\beta$ with an extent of zero, i.e. their lower and upper bounds were numerically equal, while very large confidence intervals would be expected (and indeed obtained by gmkin), because of the strong parameter correlation of $\alpha$ and $\beta$.

When fitting the DFOP model a similar pattern was observed as for the FOMC model. In general, the parameters obtained with CAKE, gmkin, KinGUII and OpenModel coincided up to three significant digits when degradation was truly biphasic, i.e. when it became slower towards the end of the data series. If the data follow an exponential decline, using the DFOP model results in an overparameterisation and parameters $k_1$, $k_2$ and *g* correlate strongly, in many cases leading to differing parameter estimates. However, the shape of the fitted curve is often hardly affected in such cases, as either parameter *g* is close to zero or close to unity, or $k_1$ and $k_2$ converge to practically equal values.

The largest differences were obtained for the parent decline curves evaluated with the hockey-stick (HS) model. Here, the exact location of the breakpoint* t*_b_ between the two different degradation rate constants often depended on the values of the starting parameters. However, when the same estimate for* t*_b_ was found, the other parameters and statistics again matched between the tools, with the exception of the $\chi ^2$ error calculations obtained with OpenModel which are still under development.

When results of model fits including metabolites were evaluated, again, parameter estimates obtained with KinGUII, gmkin and OpenModel, and, where tested, also with CAKE, were in very good agreement with deviations of less than 0.1%.

Evaluations of two datasets from water/sediment systems with a two-compartment model for the parent compound were compared as well. Initial fits were ill-determined as one of the pathways was found to be negligible. Nevertheless deviations between the remaining parameter estimates between ModelMaker, KinGUII and gmkin were less than 0.1%. For an unknown reason, parameter estimates obtained with OpenModel deviated more strongly in some cases. Subsequent fits excluding the negligible pathways resulted in the same parameter estimates, except for OpenModel which produced slightly different parameter estimates.

### Statistical measures

In addition to the good agreement found for the parameter estimates, the main goodness-of-fit criterion of the FOCUS guidance, the $\chi ^2$ error level, also matched perfectly up to three significant digits between CAKE, gmkin and KinGUII in all cases where the parameter estimates were equal to three significant digits. The only exception was the $\chi ^2$ error level obtained for metabolite m1 in the FOCUS E dataset, where KinGUII takes the residual of the first observation of the metabolite at time zero into account, which is not according to the FOCUS guidance, as this initial value is fixed to zero in the fit (compare Table S24 in additional file with the test dataset results) (Additional file 1).

The $\chi ^2$ error levels obtained for the metabolites with DegKin Manager were slightly higher than those obtained with CAKE, gmkin and KinGUII, as it accounts for the time zero sampling when calculating the degrees of freedom, which is not according to the FOCUS guidance.

The calculation of the $\chi ^2$ error level introduced in OpenModel 2.4.2 is not calculated according to the FOCUS guidance. The upcoming release is anticipated to provide the possibility to obtain $\chi ^2$ error levels according to the guidance. The $\chi ^2$ error levels obtained with the three former tools however also coincided with the results obtained with the FOCUS DegKin Spreadsheet [18] and are considered correct.

Further, it was checked if the packages that gave results for a *t* test for significant difference to zero would produce consistent results. In all cases where approximately the same parameter estimates were obtained, the result of the *t* test using a confidence level of 95% was the same, i.e. the same parameters were found to be significantly different from zero.

In contrast to OpenModel and DegKin Manager, CAKE, gmkin and KinGUII also provide confidence intervals for the parameter estimates. The confidence intervals obtained with CAKE and KinGUII are based on untransformed parameters, which makes it possible that they include physically unrealistic values like negative values for rate constants or values exceeding unity for formation fractions. In contrast, the confidence intervals obtained with gmkin, when using default settings, are based on estimates for transformed parameters in order to obtain more realistic confidence intervals [19]. The confidence intervals obtained for the test datasets illustrate these differences, but no systematic comparison of the confidence intervals obtained with the different tools was made.

### Comparison with input parameters for synthetic datasets

Due to the random component added to the synthetic datasets, it can not be expected that the input parameters used for the degradation model will be exactly reproduced by the fitting procedure. However, confidence intervals were checked against these input parameters. For all evaluations without metabolites, confidence intervals obtained with KinGUII, gmkin and CAKE included the input parameters used to generate the datasets. The same check was also made for the evaluations with metabolites for the gmkin software. For all twelve synthetic datasets, these included the input parameters and were therefore consistent with them.

### Overall ranking

The results of the quantitative ranking of the software package that received the highest scores is shown in Table 3. The scores leading to this ranking are available as a spreadsheet file as Additional file 2.

For use type I, the best scores were computed for gmkin, KinGUII and CAKE. OpenModel only reached 45% of the possible score and is therefore seen as less suitable for use type I.

**Table 3:** Results of the final quantitative ranking of the preselected software packages

Software package	Use type I		Use type II	
	Rank	Total score	Rank	Total score (%)
gmkin	1	86	1	75
KinGUII	2	82	2	73
CAKE	3	79	–	–
OpenModel	4	45	4	62
mkin	–	–	3	71

Out of the 15 software packages compared with respect to the criteria for use type II, again gmkin and KinGUII were the first two, followed by the script based tool mkin, which is the technical basis for gmkin, and by OpenModel. None of the evaluated commercial tools reached a score of 50%. This can be explained mainly by the lack of available customisations for the special use case of kinetic evaluations, but also by the fact that price and availability of the source code under an Open Source licence were among the criteria.

## Discussion

Generally, a good agreement of the software packages regarding both the estimated parameters and the statistical criteria was found. The small numerical differences found for the test datasets were not relevant for practical purposes, with the exception of the hockey stick model, where several different starting parameters have to be used in order to find the best fit.

In the case of the synthetic datasets, the fitted parameter estimates were close to the input parameters. In none of the cases where the confidence intervals were checked against these input parameters, a contradiction was found.

When using DegKin Manager and the underlying software ModelMaker, care has to be taken to adjust the time span for which the model is integrated, the accuracy of the integration and the convergence tolerance in order to obtain accurate results. Besides, the $\chi ^2$ error levels calculated for metabolites in DegKin Manager do not conform to the FOCUS guidance. When using OpenModel, it is also important to adjust the time span for which the integration takes place, the accuracy of the integration and the convergence tolerance. Also, for the version of OpenModel currently available, it is recommended to calculate the $\chi ^2$ error level using an external tool like the FOCUS DegKin spreadsheet [18].

Regarding the *t* test for significant difference from zero, only small differences were noted between the tools that provided them. As mentioned above, confidence intervals given for parameter estimates were not systematically checked as the tools use different approaches to their calculation.

The overall quantitative ranking of the tools gives a good indication which tools best fulfil the defined purposes. However, the scoring system is somewhat subjective due to the limited number of evaluators involved. Also, the comparison suffers from some problems from a practical point of view. After the system of evaluation criteria had been set up and the evaluations were done, it was found that one aspect considered important from a practical point of view was not accounted for, and others may have received too little weight.

A very important practical aspect of working with kinetic evaluation software is the total time spent by the user to perform an analysis, including the time spent to study and understand the documentation (if necessary), the time to import or format the data, to set up the models, to fit them and to document the results.

This time has not been measured and could therefore not enter the evaluation. However, from the experience of the authors, using the CAKE software probably takes the least time, and using the KinGUII and OpenModel packages generally take the most time. This is not reflected in the scores shown in Table 3.

On the other hand, these rankings, even if the exact numbers should not be taken too seriously, show which packages offer the most support in the task of deriving parameters and regulatory endpoints from chemical degradation data.

For users that prefer a workflow involving scripts rather then a graphical user interface, the R package mkin, which is the technical basis of the gmkin package, also provides a viable alternative. Its ability to increase the evaluation speed for models with metabolites when a compiler is installed is inherited by the gmkin user interface. OpenModel also uses compiled model code which therefore also runs quite fast. The flexibility of mkin to adapt the graphical output is not provided by any of the other tools.

## Conclusions

A comparison of numerical results of the software tools gmkin, KinGUII, CAKE and OpenModel used for kinetic evaluations showed excellent agreement of the estimated parameters when the chosen model was suitable for the observed decline and local minima observed for the hockey stick model are avoided. Besides, good agreement was observed for the statistical parameters $\chi ^2$ error level and *t* test for the software tools gmkin, KinGUII and CAKE.

For this purpose, a set of test data was assembled that can be used to compare the numerical results for different software tools and to ensure that no unintended or erroneous behaviour is introduced in newer versions.

In the overall ranking of the software tools it was found that due to the somewhat subjective nature of the scoring procedure, there is some uncertainty regarding the exact percentage scores.

Based on these scores, gmkin, KinGUII and CAKE were identified as the software packages that are best suited for the task of regulatory evaluation of chemical degradation data for use type I.

For use type II that also has to deal with more complex degradation models and pathways that CAKE cannot handle, gmkin and KinGUII are recommended. OpenModel is also a viable alternative for this use type, if the user spends some additonal time on adjusting default integration and optimization parameters and uses an external tool for calculation of the $\chi ^2$ error level. Out of the tools with a graphical user interface, OpenModel is the most flexible one. For users that prefer to work with scripts instead of graphical user interfaces, the R package mkin is also a valid alternative.

For future software evaluations, it is recommended to include a measure for the total time needed by an average user to perform a certain kinetic evaluation into the scoring scheme.

It is the hope of the authors that the publication of test data, source code and overall rankings foster the evolution of useful and reliable software in the field [20].

## Additional files

**Additional file 1.** Numerical results of evaluations of the test datasets.

**Additional file 2.** Spreadsheet used for selection and overall ranking of the tools.

## References

[1] Stephenson MS (1977). An approach to the identification of organic compounds hazardous to the environment and human health. Ecotoxicol Environ Saf.

[2] FOCUS (2006) Guidance document on estimating persistence and degradation kinetics from environmental fate studies on pesticides in EU registration. Report of the FOCUS Work Group on Degradation Kinetics, EC Doc. Ref. Sanco/10058/2005, version 2.0, Work Group on Degradation Kinetics of FOCUS (FOrum for the Co-ordination of pesticide fate models and their USe)

[3] FOCUS (2014) Generic guidance for estimating persistence and degradation kinetics from environmental fate studies on pesticides in EU registration. Report version 1.1

[4] Görlitz L, Gao Z, Schmitt W (2011). Statistical analysis of chemical transformation kinetics using markov-chain monte carlo methods. Environ Sci Technol.

[5] Gao Z, Green JW, Vanderborght J, Schmitt W (2011). Improving uncertainty analysis in kinetic evaluations using iteratively reweighted least squares. Environ Sci Technol.

[6] Witt J, Gao Z, Meyer H. (2014). KinGUII V2.1. http://kinguii.vrbka.net/KinGUIIv2.1.zip. Accessed 15 May 2018

[7] R Core Team (2018) R: a language and environment for statistical computing. R Foundation for Statistical Computing, Vienna, Austria. R Foundation for Statistical Computing. https://www.R-project.org/. Accessed 15 May 2018

[8] Ranke J (2018) mkin 0.9.47.1: kinetic evaluation of chemical degradation data. https://CRAN.R-project.org/package=mkin. Accessed 15 May 2018 10.1186/s12302-018-0145-1 PMC5960009 29805951

[9] Tessella Technology and Consulting (2018) CAKE 3.3: Computer Assisted Kinetic Evaluation. https://showcase.tessella.com/products/cake/. Accessed 15 May 2018

[10] Ranke J (2017) gmkin 0.6.8: graphical user interface for fitting kinetic models to chemical degradation data. https://pkgdown.jrwb.de/gmkin. Accessed 15 May 2018

[11] Crout N (2016) OpenModel 2.4.2. http://openmodel.info. Accessed 15 May 2018

[12] Honti M, Fenner K (2015). Deriving persistence indicators from regulatory water-sediment studies—opportunities and limitations in oecd 308 data. Environ Sci Technol.

[13] Rocke DM, Lorenzato S (1995). A two-component model for measurement error in analytical chemistry. Technometrics.

[14] Ranke J (2018) Synthetic datasets for one parent compound with two metabolites. https://pkgdown.jrwb.de/mkin/reference/synthetic_data_for_UBA.html. Accessed 15 May 2018

[15] Soetaert K, Petzoldt T (2010). Inverse modelling, sensitivity and monte carlo analysis in R using package FME. J Stat Softw.

[16] Gustafson DI, Holden LR (1990). Nonlinear pesticide dissipation in soil: A new model based on spatial variability. Environ Sci Technol.

[17] Ranke J (2011) kinfit—routines for fitting kinetic models to chemical degradation data. R package vignette. https://cgit.jrwb.de/kinfit/plain/inst/doc/kinfit.pdf?id=3a6b9f52c74d6ef88a8d32c50e42864b3f251719

[18] FOCUS Degradation Kinetics Spreadsheet (facilitating Analysis for Parent Compounds) FOCUS DEGKIN V2. (2007). https://esdac.jrc.ec.europa.eu/projects/degradation-kinetics-software

[19] Ranke J, Lehmann R (2012) Parameter reliability in kinetic evaluation of environmental metabolism data—assessment and the influence of model specification. Poster presented at the 6th SETAC World congress, 20–24 May 2012, Berlin

[20] Lie K-A (2017) On holden’s seven guidelines for scientific computing and development of open-source community software. preprint

