# Supplementary material for: Comparison of software tools for kinetic evaluation of chemical degradation data
Source: Environ Sci Eur. 2018 May 18;30(1):17. doi: 10.1186/s12302-018-0145-1 (PMC5960009; doi:10.1186/s12302-018-0145-1)
Supplement: Supplementary file 1 — Additional file 1. Numerical results of evaluations of the test datasets. [file 12302_2018_145_MOESM1_ESM.pdf]

Supporting information to: Comparison of software tools for kinetic evaluation of chemical degradation data

PD Dr. Johannes Ranke

Wissenschaftlicher Berater
Kronacher Str. 12
79639 Grenzach-Wyhlen
Germany

johannes.ranke@jrwb.de

Sponsor: German Environment Agency (UBA)
Wörlitzer Platz 1, 06844 Dessau-Roßlau, Germany

Report ID: jrwb-116

Draft from 10 April 2018

40 pages

Contents

Title page 1

Contents 2

Introduction 4

General remarks 4

Results for test datasets from the FOCUS guidance 5
- FOCUS A . . . 6
- FOCUS C . . . 10
- FOCUS D . . . 13
- FOCUS E . . . 15

Results for synthetic datasets 18
- SFO_lin_a . . . 19
- SFO_lin_b . . . 21
- SFO_lin_c . . . 23
- DFOP_lin_a . . . 25
- DFOP_lin_b . . . 27
- DFOP_lin_c . . . 29
- SFO_par_a . . . 31
- SFO_par_b . . . 32
- SFO_par_c . . . 33
- DFOP_par_a . . . 34
- DFOP_par_b . . . 35
- DFOP_par_c . . . 36

Results for experimental test datasets 37
- Test data UBA 2014 River . . . 37
- Test data UBA 2014 Pond . . . 38
- Test data UBA 2014 Soil . . . 39

References 40

List of Tables

S1 Parameter starting values used where no defaults were available 4
S2 FOCUS dataset A 6
S3 Results for FOCUS A evaluated with SFO 6
S4 Relative deviations for FOCUS A evaluated with SFO 6
S5 Results for FOCUS A evaluated with FOMC 7
S6 Relative deviations for A FOMC evaluated with FOMC 7
S7 Results for FOCUS A evaluated with DFOP 8
S8 Relative deviations for FOCUS A evaluated with DFOP 8
S9 Results for FOCUS A evaluated with HS 8
S10 Relative deviations for FOCUS A evaluated with HS 9
S11 FOCUS dataset C 10
S12 Results for FOCUS C evaluated with SFO 10
S13 Relative deviations for FOCUS C evaluated with SFO 10
S14 Results for FOCUS C evaluated with FOMC 11
S15 Relative deviations for FOCUS C evaluated with FOMC 11
S16 Results for FOCUS C evaluated with DFOP 11
S17 Relative deviations for FOCUS C evaluated with DFOP 11
S18 Results for FOCUS C evaluated with HS 12
S19 Relative deviations for FOCUS C evaluated with HS 12
S20 FOCUS dataset D 13
S21 Results for FOCUS D evaluated with SFO-SFO 13
S22 Relative deviations for FOCUS D evaluated with SFO-SFO 14
S23 FOCUS dataset E 15
S24 Results for FOCUS E evaluated with SFO-SFO 15
S25 Relative deviations for FOCUS D evaluated with SFO-SFO 16
S26 Results for FOCUS E evaluated with DFOP-SFO 16
S27 Relative deviations for FOCUS E evaluated with DFOP-SFO 17
S28 Synthetic dataset SFO_lin_a 19
S29 Results with confidence intervals for SFO_lin_a evaluated with SFO 19
S30 Results with confidence intervals for SFO_lin_a evaluated with FOMC 19
S31 Results with confidence intervals for SFO_lin_a evaluated with DFOP 20
S32 Results with confidence intervals for SFO_lin_a evaluated with HS 20
S33 Results for SFO_lin_a evaluated with SFO_lin 20

S34 Synthetic dataset SFO_lin_b 21
S35 Results with confidence intervals for SFO_lin_b evaluated with SFO 21
S36 Results with confidence intervals for SFO_lin_b evaluated with FOMC 22
S37 Results with confidence intervals for SFO_lin_b evaluated with DFOP 22
S38 Results with confidence intervals for SFO_lin_b evaluated with HS 22
S39 Results for SFO_lin_b evaluated with SFO_lin 22
S40 Synthetic dataset SFO_lin_c 23
S41 Results with confidence intervals for SFO_lin_c evaluated with SFO 23
S42 Results with confidence intervals for SFO_lin_c evaluated with FOMC 23
S43 Results with confidence intervals for SFO_lin_c evaluated with DFOP 24
S44 Results with confidence intervals for SFO_lin_c evaluated with HS 24
S45 Results for SFO_lin_c evaluated with SFO_lin 24
S46 Synthetic dataset DFOP_lin_a 25
S47 Results with confidence intervals for DFOP_lin_a evaluated with SFO 25
S48 Results with confidence intervals for DFOP_lin_a evaluated with FOMC 25
S49 Results with confidence intervals for DFOP_lin_a evaluated with DFOP 26
S50 Results with confidence intervals for DFOP_lin_a evaluated with HS 26
S51 Results for DFOP_lin_a evaluated with SFO_lin 26
S52 Synthetic dataset DFOP_lin_b 27
S53 Results with confidence intervals for DFOP_lin_b evaluated with SFO 27
S54 Results with confidence intervals for DFOP_lin_b evaluated with FOMC 27
S55 Results with confidence intervals for DFOP_lin_b evaluated with DFOP 28
S56 Results with confidence intervals for DFOP_lin_b evaluated with HS 28
S57 Results for DFOP_lin_b evaluated with SFO_lin 28
S58 Synthetic dataset DFOP_lin_c 29
S59 Results with confidence intervals for DFOP_lin_c evaluated with SFO 29
S60 Results with confidence intervals for DFOP_lin_c evaluated with FOMC 29
S61 Results with confidence intervals for DFOP_lin_c evaluated with DFOP 30
S62 Results with confidence intervals for DFOP_lin_c evaluated with HS 30
S63 Results for DFOP_lin_c evaluated with SFO_lin 30
S64 Synthetic dataset SFO_par_a 31
S65 Results for SFO_par_a evaluated with SFO_par 31
S66 Synthetic dataset SFO_par_b 32
S67 Results for SFO_par_b evaluated with SFO_par 32

S68 Synthetic dataset SFO_par_c 33
S69 Results for SFO_par_c evaluated with SFO_par 33
S70 Synthetic dataset DFOP_par_a 34
S71 Results for DFOP_par_a evaluated with DFOP_par 34
S72 Synthetic dataset DFOP_par_b 35
S73 Results for DFOP_par_b evaluated with DFOP_par 35
S74 Synthetic dataset DFOP_par_c 36
S75 Results for DFOP_par_c evaluated with DFOP_par 36
S76 Experimental test dataset UBA_2014_WS_river 37
S77 Results for UBA_2014_WS_river 37
S78 Experimental test dataset UBA_2014_WS_pond 38
S79 Results for UBA_2014_WS_pond 38
S80 Experimental test dataset UBA_2014_soil 39
S81 Results for UBA_2014_soil 39

Introduction

This document contains annotated results of the evaluation of test datasets assembled in the course of two projects carried out by the author for the German Environment Agency (UBA). A first version of the results of these evaluations using different software tools has been performed in 2014 in Project No 27452. An update of this comparison with the current versions has been commissioned to JR in Project No 92570. This document is being published as supporting information to a manuscript about a more general software comparison (Ranke et al., 2018). Further updates and/or extensions of this document may be published elsewhere.

General remarks

The datasets were evaluated with DegKin Manager in version 1.0, together with ModelMaker 4.0.0, published in 2000, which is used by DegKin Manager for the actual integration and optimisation. CAKE version 3.2, KinGUII version 2.1 with Version number 2.2014.224.1704, OpenModel Version 2.4.2 and mkin version 0.9.47.1.

Some software settings were adjusted. For ModelMaker (used by DegKin Manager) and OpenModel, the stop value for the output time was set to the last sampling time in the respective test dataset. This is not necessary for the other software packages.

For the integration of kinetic models with ModelMaker, Runge-Kutta integration with 200 output points was used, the integration accuracy was set to 0.001 and constant error scaling was specified. For the automatic steplength calculation with OpenModel, an error factor of at least 1e-5 was specified.

For the optimisation, the settings that were predefined in the model files supplied by DegKin manager were generally not changed. For the termination criterion, the value for the fractional change was 0.01 in some model files and 0.001 in others. In OpenModel, the change threshold for the convergence was set to 1e-5 and the maximum number of iterations was set to 200.

No weighting methods were enabled. For parameter starting values, the values predefined by the software packages were used. If not available, the values given in Table S1 were used.

Table S1: Parameter starting values used where no defaults were available

Parameter names	
parent_0	100
k values	0.1
alpha	1
beta	10
k1	0.1
k2	0.01
g	0.5
tb	5

No χ^2 error level values are shown for OpenModel, as they are known not to be calculated according to the FOCUS recommendations in OpenModel 2.4.2.

Results for test datasets from the FOCUS guidance

FOCUS datasets A, C, D and F that are treated here have already been evaluated with a number of software packages in the original FOCUS guidance from 2006 (FOCUS, 2006, 2014). These results were discussed in the course of the validation of the kinfit package, which is a predecessor of the mkin package, and the median of the parameters obtained with the different packages was calculated. As no χ^2 error level values were reported in the FOCUS guidance, these values were calculated at the time with KinGUI version 1 for the kinfit package vignette (Ranke, 2011).

In this section, the median parameter values from the FOCUS guidance and the χ^2 error level values calculated with KinGUI version 1 are compared to the values obtained with DegKin Manager, KinGUII, CAKE, OpenModel and mkin.

FOCUS A

Table S2: FOCUS dataset A

Time	parent
0	101.24
3	99.27
7	90.11
14	72.19
30	29.71
62	5.98
90	1.54
118	0.39

Table S3: Results for FOCUS A evaluated with SFO

Result	Reference	DegKinM	KinGUII	CAKE	OpenModel	mkin
parent_0	109.15	109.12	109.2	109.2	109.14	109.2
k_parent	0.0372	0.0371	0.0372	0.0372	0.0372	0.03722
err [%]	8.39	8.38	8.39	8.39	-	8.385
DT50	18.63	18.68	18.62	18.6	-	18.62
DT90	61.9	62.06	61.87	61.9	-	61.87

Table S4: Relative deviations for FOCUS A evaluated with SFO

Result	DegKinM [%]	KinGUII [%]	CAKE [%]	OpenModel [%]	mkin [%]
parent_0	0.03	0.05	0.05	0.01	0.05
k_parent	0.27	< 0.01	< 0.01	< 0.01	0.05
err [%]	0.12	< 0.01	< 0.01	-	0.06
DT50	0.27	0.05	0.16	-	0.05
DT90	0.26	0.05	< 0.01	-	0.05

FOCUS dataset A closely follows a simple exponential decline as described by the SFO model. Differences of the results of the SFO fit shown in Table S3 from the reference were less than 0.1% (Table S4).

Table S5: Results for FOCUS A evaluated with FOMC

Result	Reference	DegKinM	KinGUII	CAKE	OpenModel	mkin
parent_0	109.2	109.2	109.2	109.2	109.3	109.2[b]
alpha	19000	99.52	8236	1e+05[a]	56.3	74160[b]
beta	730000	2661.9	220000	2700000[a]	1495	1993000[b]
err [%]	9.31	9.05	8.94	8.94	-	8.943
DT50	18.62	18.61	18.62	18.6	-	18.62
DT90	61.87	62.31	61.87	61.9	-	61.87

[a] No confidence intervals and t-tests available, covariance matrix could not be estimated
[b] No convergence

Table S6: Relative deviations for A FOMC evaluated with FOMC

Result	DegKinM [%]	KinGUII [%]	CAKE [%]	OpenModel [%]	mkin [%]
parent_0	< 0.01	< 0.01	< 0.01	0.09	< 0.01
alpha	99.48	56.65	426.32	99.70	290.32
beta	99.64	69.86	269.86	99.80	173.01
err [%]	2.79	3.97	3.97	-	3.94
DT50	0.05	< 0.01	0.11	-	< 0.01
DT90	0.71	< 0.01	0.05	-	< 0.01

As FOCUS dataset A is well described by the two-parameter SFO model, the FOMC model with its three parameters is already overparameterised. This leads to a lack of convergence of the FOMC fit to this dataset in mkin. Also, the covariance matrix used for describing parameter uncertainty can not be estimated by CAKE, due to the large correlation of parameters alpha and beta in this fit (Table S5).

The large relative deviations between the tools found for the alpha and beta parameters for this dataset also reflect this overparameterisation, while the resulting DT50 and DT90 values show good agreement (Table S6).

The DFOP model (Table S7) and the HS model (Table S9) are also overparameterised. With DegKin Manger, no results could be obtained because the fits terminated with the error message "singular curvature matrix encountered".

Table S7: Results for FOCUS A evaluated with DFOP

Result	Reference	DegKinM	KinGUII	CAKE	OpenModel	mkin
parent_0	109.15	NA[a]	109.2	109.2[b]	108.2	109.2[b]
k1	0.0372	NA[a]	0.0372	0.0372[b]	0.032	0.03722[b]
k2	0.0372	NA[a]	0.0372	0.0372[b]	0.032	0.03722[b]
g	0.54	NA[a]	1	0.575[b]	1	0.6449[b]
err [%]	9.66	NA[a]	9.66	9.66	-	9.66
DT50	18.62	NA[a]	18.62	18.6	-	18.62
DT90	61.87	NA[a]	61.87	61.9	-	61.87

[a] No results available because the fit did not successfully terminate
[b] No confidence intervals and t-tests available, covariance matrix could not be estimated

Table S8: Relative deviations for FOCUS A evaluated with DFOP

Result	DegKinM [%]	KinGUII [%]	CAKE [%]	OpenModel [%]	mkin [%]
parent_0	-	0.05	0.05	0.87	0.05
k1	-	< 0.01	< 0.01	13.98	0.05
k2	-	< 0.01	< 0.01	13.98	0.05
g	-	85.19	6.48	85.19	19.43
err [%]	-	< 0.01	< 0.01	-	< 0.01
DT50	-	< 0.01	0.11	-	< 0.01
DT90	-	< 0.01	0.05	-	< 0.01

While DT50 and DT90 values found by the different tools for the HS model, the different χ^2 error level, DT50 and DT90 values found by KinGUII and OpenModel show that a different solution is found by these tools. KinGUII finds the solution with the lower χ^2 error level of 1.68% when a value of 12 or greater is used as starting value for the break point tb of the HS curve. For OpenModel, a very similar minimum is found when starting values of 12, 0.01 and 0.05 are used for tb, k1 and k2, respectively.

Table S9: Results for FOCUS A evaluated with HS

Result	Reference	DegKinM	KinGUII	CAKE	OpenModel	mkin
parent_0	102.3	NA[a]	101.2	102.3[b]	101.4	102.3[b]
k1	0.0167	NA[a]	0.0066	0.0167[b]	0.0024	0.01672[b]
k2	0.0544	NA[a]	0.0462	0.0545[b]	0.043	0.05445[b]
tb	10.91	NA[a]	5.97	10.9[b]	2.48	10.91[b]
err [%]	4.11	NA[a]	4.1	1.68	-	1.678
DT50	20.29	NA[a]	20.12	20.3	-	20.29
DT90	49.86	NA[a]	54.95	49.9	-	49.85

[a] No results available because the fit did not successfully terminate
[b] No confidence intervals and t-tests available, covariance matrix could not be estimated

Table S10: Relative deviations for FOCUS A evaluated with HS

Result	DegKinM [%]	KinGUII [%]	CAKE [%]	OpenModel [%]	mkin [%]
parent_0	-	1.08	< 0.01	0.88	< 0.01
k1	-	60.48	< 0.01	85.63	0.12
k2	-	15.07	0.18	20.96	0.09
tb	-	45.28	0.09	77.27	< 0.01
err [%]	-	0.24	59.12	-	59.17
DT50	-	0.84	0.05	-	< 0.01
DT90	-	10.21	0.08	-	0.02

FOCUS C

For this dataset the results obtained with the different tools were very similar, with the exception of the DT90 value obtained with DegKin for the Hockey Stick model.

Table S11: FOCUS dataset C

Time	parent
0	85.1
1	57.9
3	29.9
7	14.6
14	9.7
28	6.6
63	4
91	3.9
119	0.6

Table S12: Results for FOCUS C evaluated with SFO

Result	Reference	DegKinM	KinGUII	CAKE	OpenModel	mkin
parent_0	82.49	82.53	82.49	82.49	82.51	82.49
k_parent	0.3062	0.3062	0.3061	0.3061	0.3064	0.3061
err [%]	15.85	15.85	15.85	15.9	-	15.85
DT50	2.265	2.26	2.265	2.27	-	2.265
DT90	7.52	7.52	7.52	7.52	-	7.523

Table S13: Relative deviations for FOCUS C evaluated with SFO

Result	DegKinM [%]	KinGUII [%]	CAKE [%]	OpenModel [%]	mkin [%]
parent_0	0.05	< 0.01	< 0.01	0.02	< 0.01
k_parent	< 0.01	0.03	0.03	0.07	0.03
err [%]	< 0.01	< 0.01	0.32	-	< 0.01
DT50	0.22	< 0.01	0.22	-	< 0.01
DT90	< 0.01	< 0.01	< 0.01	-	0.04

Deviations between the tools were less than 1%.

Table S14: Results for FOCUS C evaluated with FOMC

Result	Reference	DegKinM	KinGUII	CAKE	OpenModel	mkin
parent_0	85.88	82.53	85.88	85.87	85.88	85.87
alpha	1.05	1.04	1.05	1.05	1.054	1.053
beta	1.92	1.89	1.92	1.92	1.918	1.917
err [%]	6.66	6.66	6.66	6.66	-	6.657
DT50	1.79	1.79	1.79	1.79	-	1.785
DT90	15.15	15.39	15.15	15.2	-	15.15

Table S15: Relative deviations for FOCUS C evaluated with FOMC

Result	DegKinM [%]	KinGUII [%]	CAKE [%]	OpenModel [%]	mkin [%]
parent_0	3.90	< 0.01	0.01	< 0.01	0.01
alpha	0.95	< 0.01	< 0.01	0.38	0.29
beta	1.56	< 0.01	< 0.01	0.10	0.16
err [%]	< 0.01	< 0.01	< 0.01	-	0.05
DT50	< 0.01	< 0.01	< 0.01	-	0.28
DT90	1.58	< 0.01	0.33	-	< 0.01

Deviations between the tools were less than 1% for the FOMC model.

Table S16: Results for FOCUS C evaluated with DFOP

Result	Reference	DegKinM	KinGUII	CAKE	mkin
parent_0	-	85	85	85	85
k1	-	0.4585	0.4596	0.4596	0.4596
k2	-	0.0178	0.0178	0.0179	0.01785
g	-	0.8539	0.854	0.8539	0.8539
err [%]	2.66	2.66	2.66	2.66	2.661
DT50	-	2	1.887	1.89	1.887
DT90	-	22	21.25	21.3	21.25

Table S17: Relative deviations for FOCUS C evaluated with DFOP

Result	DegKinM [%]	KinGUII [%]	CAKE [%]	mkin [%]
parent_0	-	-	-	-
k1	-	-	-	-
k2	-	-	-	-
g	-	-	-	-
err [%]	< 0.01	< 0.01	< 0.01	0.04
DT50	-	-	-	-
DT90	-	-	-	-

Where a comparison with the reference was possible, deviations between the tools were less than 1% for the DFOP model.

Table S18: Results for FOCUS C evaluated with HS

Result	Reference	DegKinM	KinGUII	CAKE	mkin
parent_0	84.5	84.5	84.5	84.5	84.5
k1	0.3562	0.3553	0.3562	0.3562	0.3562
k2	0.0226	0.0224	0.0227	0.0226	0.02266
tb	5.15	5.16	5.15	5.15	5.153
err [%]	4.69	4.7	4.7	4.7	4.696
DT50	1.95	1.95	1.95	1.95	1.946
DT90	25.77	21.01	25.78	25.8	25.78

Table S19: Relative deviations for FOCUS C evaluated with HS

Result	DegKinM [%]	KinGUII [%]	CAKE [%]	mkin [%]
parent_0	< 0.01	< 0.01	< 0.01	< 0.01
k1	0.25	< 0.01	< 0.01	< 0.01
k2	0.88	0.44	< 0.01	0.27
tb	0.19	< 0.01	< 0.01	0.06
err [%]	0.21	0.21	0.21	0.13
DT50	< 0.01	< 0.01	< 0.01	0.21
DT90	18.47	0.04	0.12	0.04

Deviations between the tools were less than 1% for the HS model, with the exception of the DT90 calculated by DegKin Manager for the DT90 value, which appears to be erroneous in this case. Calculating the DT90 from the parameters found by DegKin Manager using the formula from the FOCUS guidance yields 26.1.

FOCUS D

Results for dataset FOCUS D are shown for the SFO-SFO model (SFO used for parent and metabolite).

Table S20: FOCUS dataset D

Time	parent	m1
0	99.46	0
0	102.04	0
1	93.5	4.84
1	92.5	5.64
3	63.23	12.91
3	68.99	12.96
7	52.32	22.97
7	55.13	24.47
14	27.27	41.69
14	26.64	33.21
21	11.5	44.37
21	11.64	46.44
35	2.85	41.22
35	2.91	37.95
50	0.69	41.19
50	0.63	40.01
75	0.05	40.09
75	0.06	33.85
100		31.04
100		33.13
120		25.15
120		33.31

Table S21: Results for FOCUS D evaluated with SFO-SFO

Result	DegKinM	KinGUII	CAKE	OpenModel	mkin	Median
parent_0	99.6	99.6	99.6	99.6	99.6	99.6
k_parent	0.0987	0.0987	0.0987	0.0987	0.0987	0.0987
k_m1	0.0053	0.0053	0.0053	0.0053	0.005261	0.0053
f_parent_m1	0.5145	0.5145	0.5145	0.5145	0.5145	0.5145
err parent [%]	6.46	6.46	6.46	-	6.459	6.46
err m1 [%]	4.95	4.69	4.69	-	4.69	4.69
err all [%]	-	6.4	6.4	-	6.398	6.4
DT50 parent	7.02	7.02	7.02	-	7.023	7.02
DT90 parent	23.33	23.33	23.3	-	23.33	23.33
DT50 m1	131.8	131.7	132	-	131.8	131.8
DT90 m1	437.7	437.6	438	-	437.7	437.7

Table S22: Relative deviations for FOCUS D evaluated with SFO-SFO

Result	DegKinM [%]	KinGUII [%]	CAKE [%]	OpenModel [%]	mkin [%]
parent_0	< 0.01	< 0.01	< 0.01	< 0.01	< 0.01
k_parent	< 0.01	< 0.01	< 0.01	< 0.01	< 0.01
k_m1	< 0.01	< 0.01	< 0.01	< 0.01	0.74
f_parent_m1	< 0.01	< 0.01	< 0.01	< 0.01	< 0.01
err parent [%]	< 0.01	< 0.01	< 0.01	-	0.02
err m1 [%]	5.54	< 0.01	< 0.01	-	< 0.01
err all [%]	-	< 0.01	< 0.01	-	0.03
DT50 parent	< 0.01	< 0.01	< 0.01	-	0.04
DT90 parent	< 0.01	< 0.01	0.13	-	< 0.01
DT50 m1	< 0.01	0.08	0.15	-	< 0.01
DT90 m1	< 0.01	0.02	0.07	-	< 0.01

Differences between the results and the median which was used as reference here are shown in Table S22 and are smaller than 1%, with the exception of the χ^2 error level for metabolite m1, where DegKin Manager takes the sampling at t=0 into account for calculating the degrees of freedom, while KinGUII, CAKE and mkin disregard this value, which is fixed to zero, in accordance with the FOCUS guidance (FOCUS, 2014, p. 89/90, 166).

Note that in order to obtain the values listed in S21 with DegKin Manager, the accuracy value for the integration was reduced to a value of 1e-8.

FOCUS E

Results for dataset FOCUS E are shown for the SFO-SFO model as well as the DFOP-SFO model where DFOP is used for the parent compound.

Table S23: FOCUS dataset E

Time	parent	m1
0	85.1	1.1
1	57.9	20
3	29.9	34
7	14.6	40.2
14	9.7	35.2
28	6.6	27.6
63	4	14.9
91	3.9	12.5
119	0.6	8.8

Table S24: Results for FOCUS E evaluated with SFO-SFO

Result	DegKinM	KinGUII	CAKE	OpenModel	mkin	Median
parent_0	84.75	84.74	84.74	84.75	84.74	84.74
k_parent	0.352	0.352	0.352	0.3521	0.352	0.352
k_m1	0.0183	0.0182	0.0183	0.0183	0.01825	0.0183
f_parent_m1	0.5658	0.5658	0.5658	0.5658	0.5658	0.5658
err parent [%]	16.59	16.59	16.6	-	16.59	16.59
err m1 [%]	11.7	11.67	11	-	10.95	11.335
err all [%]	-	15.88	15.4	-	15.44	15.44
DT50 parent	1.97	1.97	1.97	-	1.969	1.97
DT90 parent	6.54	6.54	6.54	-	6.542	6.54
DT50 m1	37.97	37.99	38	-	37.99	37.99
DT90 m1	126.1	126.2	126	-	126.2	126.15

Table S25: Relative deviations for FOCUS D evaluated with SFO-SFO

Result	DegKinM [%]	KinGUII [%]	CAKE [%]	OpenModel [%]	mkin [%]
parent_0	0.01	< 0.01	< 0.01	0.01	< 0.01
k_parent	< 0.01	< 0.01	< 0.01	0.03	< 0.01
k_m1	< 0.01	0.55	< 0.01	< 0.01	0.27
f_parent_m1	< 0.01	< 0.01	< 0.01	< 0.01	< 0.01
err parent [%]	< 0.01	< 0.01	0.06	-	< 0.01
err m1 [%]	3.22	2.96	2.96	-	3.40
err all [%]	-	2.85	0.26	-	< 0.01
DT50 parent	< 0.01	< 0.01	< 0.01	-	0.05
DT90 parent	< 0.01	< 0.01	< 0.01	-	0.03
DT50 m1	0.05	< 0.01	0.03	-	< 0.01
DT90 m1	0.04	0.04	0.12	-	0.04

Differences between the results and the median which was used as reference here are shown in Table S25 and are smaller than 1%, with the exception of the χ^2 error level for metabolite m1, where DegKin Manager takes the residual at t=0 into account in the calculation which is against the FOCUS recommendation. KinGUII uses the sampling at time 0 into account for the degrees of freedom, because it has a residue greater than zero, which also not according to the FOCUS recommendation. Current versions of CAKE and mkin handle this case according to the FOCUS guidance. For KinGUII, this can be seen in the source code of the underlying KineticEval package current at the time of this writing (link to source code at github). Here, only values at time zero that are zero are filtered out, as in mkin versions before version 0.9-33 which introduced the code currently used in mkin for this purpose (link to source code at github). However, also values different from zero occurring at time zero should be filtered out if the respective initial value is fixed to zero (FOCUS, 2014, p. 90,166).

Table S26: Results for FOCUS E evaluated with DFOP-SFO

Result	DegKinM	KinGUII	CAKE	OpenModel	mkin	Median
parent_0	85.86	85.86	85.86	85.86	85.86	85.86
k1	0.5022	0.5022	0.5022	0.5022	0.5022	0.5022
k2	0.0183	0.0183	0.0183	0.0183	0.0183	0.0183
g	0.8424	0.8424	0.8424	0.8423	0.8424	0.8424
k_m1	0.0192	0.0192	0.0192	0.0192	0.01919	0.0192
f_parent_m1	0.6083	0.6083	0.6083	0.6084	0.6083	0.6083
err parent [%]	3.7	3.7	3.7	-	3.695	3.7
err m1 [%]	6.28	6.21	5.72	-	5.72	5.965
err all [%]	-	5.6	5.38	-	5.383	5.383
DT50 parent	2	1.76	1.76	-	1.764	1.762
DT90 parent	25	24.86	24.9	-	24.86	24.88
DT50 m1	36.12	36.12	36.1	-	36.12	36.12
DT90 m1	120	120	120	-	120	120

Table S27: Relative deviations for FOCUS E evaluated with DFOP-SFO

Result	DegKinM [%]	KinGUII [%]	CAKE [%]	OpenModel [%]	mkin [%]
parent_0	< 0.01	< 0.01	< 0.01	< 0.01	< 0.01
k1	< 0.01	< 0.01	< 0.01	< 0.01	< 0.01
k2	< 0.01	< 0.01	< 0.01	< 0.01	< 0.01
g	< 0.01	< 0.01	< 0.01	0.01	< 0.01
k_m1	< 0.01	< 0.01	< 0.01	< 0.01	0.05
f_parent_m1	< 0.01	< 0.01	< 0.01	0.02	< 0.01
err parent [%]	< 0.01	< 0.01	< 0.01	-	0.14
err m1 [%]	5.28	4.11	4.11	-	4.11
err all [%]	-	4.03	0.06	-	< 0.01
DT50 parent	13.51	0.11	0.11	-	0.11
DT90 parent	0.48	0.08	0.08	-	0.08
DT50 m1	< 0.01	< 0.01	0.06	-	< 0.01
DT90 m1	< 0.01	< 0.01	< 0.01	-	< 0.01

Again, differences between the results and the median obtained from the different tools were less than 1% with the exception of the χ^2 error level for metabolite m1. DegKin Manager and KinGUII do not provide χ^2 error level values according to the FOCUS guidance to the knowledge of the author.

Results for synthetic datasets

A graphical representation of the models used for the generation of the synthetic datasets is shown in Figure 1.

Figure 1: Graphical schemes of the degradation models used for the generation of the synthetic datasets

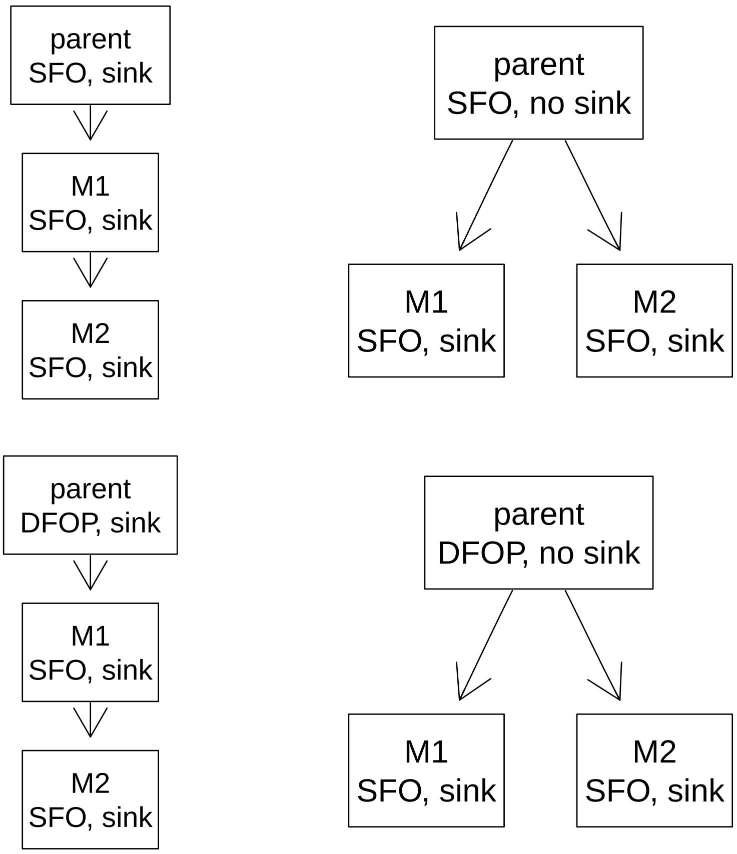

For the evaluations of the synthetic datasets, confidence intervals are reported in the following tables for the parameter estimates from the parent only evaluations using the SFO, FOMC, DFOP and HS models. This makes it possible to check if the confidence intervals include the parameters that were used in the generation of the data. The latter are shown in the column "Input" in the result tables.

For the coupled fits, confidence intervals obtained with mkin are shown.

Table S28: Synthetic dataset SFO_lin_a

Time	parent	M1	M2
0	101.5		
0	101.2		
1	53.9	36.4	
1	47.5	37.4	4.8
3	10.4	34.3	20.9
3	7.6	39.8	19.3
7	1.1	15.1	42
7	0.3	17.8	43.1
14		5.8	49.4
14	3.5	1.2	44.3
28			34.6
28	3.2		33
60		0.5	18.8
60			17.6
90	0.6		10.6
90		3.2	10.8
120		1.5	9.8
120	3.5	0.6	3.3

SFO_lin_a

Table S29: Results with confidence intervals for SFO_lin_a evaluated with SFO

Result	Input	KinGUII			CAKE			mkin		
		Estimate	From	To	Estimate	From	To	Estimate	From	To
parent_0	100	101.9	98.1	105.7	101.9	97.6	106.2	101.9	97.58	106.2
k_parent	0.7	0.731	0.66	0.801	0.731	0.651	0.811	0.7308	0.6549	0.8154
err [%]	-	8.64	-	-	8.64	-	-	8.637	-	-

Table S30: Results with confidence intervals for SFO_lin_a evaluated with FOMC

Result	Input	KinGUII			CAKE			mkin		
		Estimate	From	To	Estimate	From	To	Estimate	From	To
parent_0	100	101.9	97.8	105.9	101.9	-	-	101.9	97.23	106.5
alpha	-	20000	-1e+08	1e+08	5.2e+09	-	-	151400	0	Inf
beta	-	27000	-1e+08	1e+08	7e+09	-	-	207200	0	Inf
err [%]	-	9.21	-	-	9.21	-	-	9.212	-	-

Table S31: Results with confidence intervals for SFO_lin_a evaluated with DFOP

Result	Input	KinGUII			CAKE			mkin		
		Estimate	From	To	Estimate	From	To	Estimate	From	To
parent_0	100	102	98.1	106	101.9	96.8	106.9	102	-	-
k1	-	0.754	0.659	0.848	0.731	-	-	0.7537	-	-
k2	-	0	-0.038	0.038	0.731	-	-	1.222e-11	-	-
g	-	0.987	0.956	1.018	0.068	-84000	84000	0.9866	-	-
err [%]	-	7.81	-	-	9.65	-	-	7.812	-	-

Table S32: Results with confidence intervals for SFO_lin_a evaluated with HS

Result	Input	KinGUII			CAKE			mkin		
		Estimate	From	To	Estimate	From	To	Estimate	From	To
parent_0	100	101.9	98.4	105.4	101.9	97.7	106	101.9	-	-
k1	-	0.731	0.665	0.848	0.731	0.534	0.808	0.7309	-	-
k2	-	2.2e-14	-0.02	0.038	2.8e-13	-0.03	0.03	1.385e-11	-	-
tb	-	5.36	3.45	1.018	5.36	3.11	7.6	5.355	-	-
err [%]	-	6.41	-	-	6.41	-	-	6.406	-	-

Table S33: Results for SFO_lin_a evaluated with SFO_lin

Result	Input	DegKinM	KinGUII	OpenModel	mkin		
					Estimate	Lower	Upper
parent_0	100	102.1	102.1	102.1	102.1	98.58	105.5
k_parent	0.7	0.7374	0.7393	0.7392	0.7393	0.6775	0.8068
k_m1	0.3	0.2989	0.2992	0.2992	0.2992	0.2563	0.3492
k_m2	0.02	0.0202	0.0202	0.0202	0.02023	0.01756	0.0233
f_parent_m1	0.8	0.7684	0.7687	0.7688	0.7687	0.6638	0.8483
f_m1_m2	0.7	0.7225	0.7229	0.7229	0.7229	0.6026	0.8178
err parent [%]	-	-	8.66	-	8.66	-	-
err m1 [%]	-	-	10.58	-	10.58	-	-
err m2 [%]	-	-	3.59	-	3.586	-	-
err all [%]	-	-	8.45	-	8.454	-	-

SFO_lin_b

Table S34: Synthetic dataset SFO_lin_b

Time	parent	M1	M2
0	103.5		
0	102.8		
1	59.6	39.3	
1	44.6	41.7	5.5
3	7.9	27.1	21
3	1.3	39.8	17.3
7	1.6	13.8	43.1
7		20.1	45.7
14		10.6	55.2
14	8.1		43.2
28			33.7
28	7.4		29.9
60		1.2	19.1
60			16.2
90	1.3		11.2
90		7.5	11.6
120		3.4	15.5
120	8.2	1.3	0.2

Table S35: Results with confidence intervals for SFO_lin_b evaluated with SFO

Result	Input	KinGUII			CAKE			mkin		
		Estimate	From	To	Estimate	From	To	Estimate	From	To
parent_0	100	104.3	94.9	113.6	104.3	93.5	115.1	104.3	93.48	115.1
k_parent	0.7	0.768	0.588	0.948	0.768	0.56	0.976	0.768	0.5861	1.006
err [%]	-	18.71	-	-	18.7	-	-	18.71	-	-

Table S36: Results with confidence intervals for SFO_lin_b evaluated with FOMC

Result	Input	KinGUII			CAKE			mkin		
		Estimate	From	To	Estimate	From	To	Estimate	From	To
parent_0	100	104.3	94.2	114.4	104.3	92.4	116.2	104	92	120
alpha	-	12000	-1e+08	1e+08	680000	-1e+08	1e+08	95500	0	Inf
beta	-	16000	-1e+08	1e+08	890000	-1e+08	1e+08	124000	0	Inf
err [%]	-	19.93	-	-	20	-	-	20	-	-

Table S37: Results with confidence intervals for SFO_lin_b evaluated with DFOP

Result	Input	KinGUII			CAKE			mkin		
		Estimate	From	To	Estimate	From	To	Estimate	From	To
parent_0	100	104.4	94.7	114.2	104.3	104.3	104.3	104.4	-	-
k1	-	0.825	0.558	1.091	37.89	-	-	0.8244	-	-
k2	-	0	-0.038	0.38	0.768	-	-	7.397e-12	-	-
g	-	0.966	0.883	1.05	0	-	-	0.9664	-	-
err [%]	-	16.68	-	-	21.6	-	-	16.69	-	-

Table S38: Results with confidence intervals for SFO_lin_b evaluated with HS

Result	Input	KinGUII			CAKE			mkin		
		Estimate	From	To	Estimate	From	To	Estimate	From	To
parent_0	100	104.3	95.6	113	104.3	93.8	114.8	104.3	-	-
k1	-	0.769	0.602	0.936	0.769	0.567	0.97	0.7686	-	-
k2	-	2e-14	-0.02	0.02	3e-12	-0.03	0.03	5.412e-11	-	-
tb	-	3.87	1.73	6.02	3.87	1.28	6.46	3.872	-	-
err [%]	-	13.88	-	-	13.9	-	-	13.88	-	-

Table S39: Results for SFO_lin_b evaluated with SFO_lin

Result	Input	DegKinM	KinGUII	OpenModel	mkin		
					Estimate	Lower	Upper
parent_0	100	104.7	104.7	104.7	104.7	96.28	113.2
k_parent	0.7	0.7871	0.7897	0.7895	0.7897	0.6418	0.9717
k_m1	0.3	0.2979	0.298	0.2982	0.298	0.2052	0.4328
k_m2	0.02	0.0205	0.02056	0.02056	0.02056	0.01465	0.02884
f_parent_m1	0.8	0.7341	0.734	0.7343	0.734	0.4866	0.8894
f_m1_m2	0.7	0.75	0.7506	0.7504	0.7506	0.4157	0.9271
err parent [%]	-	13.89	18.77	-	18.77	-	-
err m1 [%]	-	27.37	26.27	-	26.27	-	-
err m2 [%]	-	9.99	8.3	-	8.305	-	-
err all [%]	-	-	19.86	-	19.86	-	-

SFO_lin_c

Table S40: Synthetic dataset SFO_lin_c

Time	parent	M1	M2
0	103.5		
0	102.8		
1	54.6	36	2.9
1	47.1	36.8	4.3
3	11.6	34.6	20.8
3	10.7	39.8	20
7	0.8	15.7	42
7	0.7	16.8	43
14		2.7	49.7
14	0.6	1.9	44.2
28			34.7
28	0.5		33.3
60			18.7
60			18.1
90			10.3
90		0.5	10.4
120		0.2	6.5
120	0.6		5.1

Table S41: Results with confidence intervals for SFO_lin_c evaluated with SFO

Result	Input	KinGUII			CAKE			mkin		
		Estimate	From	To	Estimate	From	To	Estimate	From	To
parent_0	100	103.3	100.8	105.9	103.3	100.4	106.3	103.3	100.4	106.3
k_parent	0.7	0.72	0.674	0.766	0.72	0.667	0.773	0.7197	0.6685	0.775
err [%]	-	1.73	-	-	1.73	-	-	1.729	-	-

Table S42: Results with confidence intervals for SFO_lin_c evaluated with FOMC

Result	Input	KinGUII			CAKE			mkin		
		Estimate	From	To	Estimate	From	To	Estimate	From	To
parent_0	100	103.3	100.9	105.8	103.3	100.1	106.6	103	100	110
alpha	-	5200000	5200000	5200000	1.1e+08	6e+07	1.5e+08	78600	0	Inf
beta	-	7200000	7200000	7200000	1.5e+08	1.1e+08	1.9e+08	109000	0	Inf
err [%]	-	1.87	-	-	1.87	-	-	1.87	-	-

Table S43: Results with confidence intervals for SFO_lin_c evaluated with DFOP

Result	Input	KinGUII			CAKE			mkin		
		Estimate	From	To	Estimate	From	To	Estimate	From	To
parent_0	100	103.3	100.4	106.2	103.3	-	-	103.3	-	-
k1	-	0.72	0.649	0.791	0.72	-	-	0.7232	-	-
k2	-	0.037	0.037	0.037	0.72	-	-	1.664e-10	-	-
g	-	1	0.969	1.031	0.564	-	-	0.998	-	-
err [%]	-	2.06	-	-	2.06	-	-	1.834	-	-

Table S44: Results with confidence intervals for SFO_lin_c evaluated with HS

Result	Input	KinGUII			CAKE			mkin		
		Estimate	From	To	Estimate	From	To	Estimate	From	To
parent_0	100	103.3	100.5	106.2	103.3	99.89	106.8	103.3	-	-
k1	-	0.72	0.668	0.771	0.72	0.658	0.782	0.7198	-	-
k2	-	2.2e-14	-0.09	0.09	3e-12	-0.11	0.11	9.363e-10	-	-
tb	-	7.23	-0.97	15.4	7.23	-2.7	17.1	7.233	-	-
err [%]	-	1.45	-	-	1.45	-	-	1.448	-	-

Table S45: Results for SFO_lin_c evaluated with SFO_lin

Result	Input	DegKinM	KinGUII	OpenModel	mkin		
					Estimate	Lower	Upper
parent_0	100	103.5	103.5	103.5	103.5	101	106
k_parent	0.7	0.7245	0.7261	0.7261	0.726	0.6828	0.772
k_m1	0.3	0.3024	0.3029	0.3028	0.3029	0.2713	0.3382
k_m2	0.02	0.0204	0.0205	0.0205	0.02046	0.0185	0.02263
f_parent_m1	0.8	0.7614	0.7624	0.7623	0.7624	0.6907	0.8217
f_m1_m2	0.7	0.7222	0.7218	0.7219	0.7218	0.638	0.7924
err parent [%]	-	1.39	1.79	-	1.786	-	-
err m1 [%]	-	17.89	6.25	-	6.247	-	-
err m2 [%]	-	7.15	3.1	-	3.1	-	-
err all [%]	-	-	3.93	-	3.932	-	-

DFOP_lin_a

Table S46: Synthetic dataset DFOP_lin_a

Time	parent	M1	M2
0	101.5		
0	101.2		
1	94.2	6.5	
1	87.8	7.5	1
3	72.7	2.6	3.3
3	69.8	8.1	1.7
7	56.2	6.6	10.4
7	55.3	9.3	11.5
14	36.3	7.3	20.1
14	44.3	2.7	14.9
28	25.6		16.2
28	31.9		14.6
60	11.8	1	12.8
60	12.2		11.6
90	8.8		9.2
90	4.3	3.5	9.4
120	1.3	1.6	10.1
120	8	0.7	3.5

Table S47: Results with confidence intervals for DFOP_lin_a evaluated with SFO

Result	Input	KinGUII			CAKE			mkin		
		Estimate	From	To	Estimate	From	To	Estimate	From	To
parent_0	100	92.9	86.1	99.8	92.9	85.5	100.3	92.92	85.49	100.3
k_parent	-	0.0542	0.042	0.067	0.0542	0.04	0.68	0.05416	0.04218	0.06954
err [%]	-	11.76	-	-	11.8	-	-	11.76	-	-

Table S48: Results with confidence intervals for DFOP_lin_a evaluated with FOMC

Result	Input	KinGUII			CAKE			mkin		
		Estimate	From	To	Estimate	From	To	Estimate	From	To
parent_0	100	100.4	96	104.7	100.4	95.6	105.1	100.4	95.63	105.1
alpha	-	0.989	0.708	1.27	0.989	0.68	1.3	0.9892	0.7261	1.347
beta	-	8.721	4.323	13.12	8.72	3.94	13.5	8.721	5.039	15.09
err [%]	-	4.39	-	-	4.39	-	-	4.392	-	-

Table S49: Results with confidence intervals for DFOP_lin_a evaluated with DFOP

Result	Input	KinGUII			CAKE			mkin		
		Estimate	From	To	Estimate	From	To	Estimate	From	To
parent_0	100	101.7	98	105.5	101.7	97.6	105.9	101.7	97.6	105.9
k1	0.2	0.261	0.159	0.363	0.261	0.139	0.373	0.2609	0.1699	0.4006
k2	0.02	0.0241	0.019	0.03	0.0241	0.018	0.03	0.02408	0.01864	0.03111
g	0.5	0.453	0.36	0.55	0.453	0.35	0.56	0.453	0.353	0.557
err [%]	-	2.08	-	-	2.08	-	-	2.078	-	-

Table S50: Results with confidence intervals for DFOP_lin_a evaluated with HS

Result	Input	KinGUII			CAKE			mkin		
		Estimate	From	To	Estimate	From	To	Estimate	From	To
parent_0	100	101.7	97.5	105.8	101.7	97.2	106.2	101.7	97.15	106.2
k1	-	0.117	0.09	0.144	0.117	0.088	0.147	0.1174	0.09135	0.151
k2	-	0.0283	0.23	0.034	0.028	0.023	0.034	0.02829	0.02306	0.03469
tb	-	5.07	3.52	6.63	5.07	3.37	6.78	5.073	3.626	7.099
err [%]	-	3.4	-	-	3.4	-	-	3.403	-	-

Table S51: Results for DFOP_lin_a evaluated with SFO_lin

Result	Input	DegKinM	KinGUII	OpenModel	mkin		
					Estimate	Lower	Upper
parent_0	100	101.8	101.7	101.7	101.7	98.27	105.1
k1	0.2	0.2563	0.2554	0.2554	0.2778	0.1392	0.5546
k2	0.02	0.0236	0.0236	0.0236	0.0205	0.01399	0.03005
g	0.5	0.4592	0.46	4598	0.4369	0.2225	0.6778
k_m1	0.3	0.2762	0.2778	0.2779	0.7994	0.1062	0.9926
k_m2	0.02	0.0205	0.0205	0.0205	0.2554	0.1802	0.3619
f_parent_m1	0.5	0.434	0.4369	0.4371	0.02363	0.01911	0.02921
f_m1_m2	0.7	0.8045	0.7994	0.7993	0.4599	0.3769	0.5452
err parent [%]	-	2.09	2.09	-	2.089	-	-
err m1 [%]	-	35.71	36.74	-	36.74	-	-
err m2 [%]	-	9.52	8.9	-	8.9	-	-
err all [%]	-	-	6.27	-	6.273	-	-

DFOP_lin_b

Table S52: Synthetic dataset DFOP_lin_b

Time	parent	M1	M2
0	103.5		
0	102.8		
1	99.9	9.4	
1	84.9	11.8	1.8
3	70.2		3.4
3	63.6	8.1	
7	56.7	5.3	11.6
7	54.7	11.6	14.1
14	30.2	12.2	25.8
14	49	1.6	13.9
28	21.3		15.3
28	36.1		11.6
60	7.4	1.7	13.1
60	8.4		10.3
90	9.6		9.8
90		7.8	10.2
120		3.6	15.7
120	12.7	1.4	0.4

Table S53: Results with confidence intervals for DFOP_lin_b evaluated with SFO

Result	Input	KinGUII			CAKE			mkin		
		Estimate	From	To	Estimate	From	To	Estimate	From	To
parent_0	100	93.8	84.3	103.4	93.8	83.4	104.3	93.82	83.39	104.3
k_parent	-	0.0583	0.04	0.077	0.0583	0.038	0.078	0.05833	0.04135	0.08227
err [%]	-	14.87	-	-	14.9	-	-	14.87	-	-

Table S54: Results with confidence intervals for DFOP_lin_b evaluated with FOMC

Result	Input	KinGUII			CAKE			mkin		
		Estimate	From	To	Estimate	From	To	Estimate	From	To
parent_0	100	103.1	94.4	111.7	103.1	93.5	112.6	103.1	93.5	113
alpha	-	0.772	0.393	1.15	0.772	0.35	1.19	0.7721	0.449	1.33
beta	-	5.24	0.223	10.25	5.24	-0.29	10.76	5.237	1.82	15
err [%]	-	6.81	-	-	6.81	-	-	6.81	-	-

Table S55: Results with confidence intervals for DFOP_lin_b evaluated with DFOP

Result	Input	KinGUII			CAKE			mkin		
		Estimate	From	To	Estimate	From	To	Estimate	From	To
parent_0	100	104.2	94.8	113.6	104.2	93.8	114.7	104.2	93.76	114.7
k1	0.2	0.341	0.026	0.656	0.342	-0.009	0.69	0.3412	0.1223	0.9515
k2	0.02	0.0263	0.014	0.041	0.026	0.009	0.043	0.02629	0.01399	0.04941
g	0.5	0.44	0.24	0.64	0.44	0.21	0.67	0.4396	0.2384	0.6628
err [%]	-	8.5	-	-	8.5	-	-	8.496	-	-

Table S56: Results with confidence intervals for DFOP_lin_b evaluated with HS

Result	Input	KinGUII			CAKE			mkin		
		Estimate	From	To	Estimate	From	To	Estimate	From	To
parent_0	100	104.3	95.4	113.2	100.6	90.9	110.3	104.3	94.4	114.2
k1	-	0.144	0.085	0.203	0.097	0.061	0.134	0.1436	0.09091	0.2269
k2	-	0.0308	0.018	0.044	0.025	0.007	0.043	0.03075	0.01909	0.04951
tb	-	4	1.7	6.4	8.1	2.2	14	4.047	2.108	7.768
err [%]	-	8.77	-	-	9.89	-	-	8.766	-	-

Table S57: Results for DFOP_lin_b evaluated with SFO_lin

Result	Input	DegKinM	KinGUII	OpenModel	mkin		
					Estimate	Lower	Upper
parent_0	100	104.3	104.1	104.1	104.1	96.36	111.8
k1	0.2	0.3426	0.3235	0.3241	0.3914	0.1081	1.417
k2	0.02	0.0259	0.02511	0.02516	0.01883	0.008855	0.04006
g	0.5	0.4423	0.4539	0.4534	0.714	0.05076	0.9915
k_m1	0.3	0.3753	0.3912	0.3918	0.4575	0.07159	0.9022
k_m2	0.02	0.0191	0.0188	0.0189	0.3235	0.1555	0.6731
f_parent_m1	0.5	0.6882	0.7137	0.7145	0.02511	0.01566	0.04028
f_m1_m2	0.7	0.4739	0.4577	0.4573	0.454	0.2988	0.6186
err parent [%]	-	7.72	8.39	-	8.389	-	-
err m1 [%]	-	51	47.1	-	47.1	-	-
err m2 [%]	-	15.21	15.92	-	15.92	-	-
err all [%]	-	-	15.06	-	15.06	-	-

DFOP_lin_c

Table S58: Synthetic dataset DFOP_lin_c

Time	parent	M1	M2
0	103.5		
0	102.8		
1	98.9	4.7	
1	85.4	4.9	0.6
3	71.3	6.7	3.2
3	66.3	8.1	2.9
7	56.3	7.3	9.8
7	55.2	8	10.1
14	36.4	4.3	17.5
14	44.2	3.5	15.4
28	26.5	0.5	16.6
28	30.9	0.9	15.9
60	13.8	0.6	12.7
60	14		12.3
90	8.4		8.9
90	7.3	0.8	9
120	3.9	0.4	6.8
120	5.2	0.3	5.3

Table S59: Results with confidence intervals for DFOP_lin_c evaluated with SFO

Result	Input	KinGUII			CAKE			mkin		
		Estimate	From	To	Estimate	From	To	Estimate	From	To
parent_0	100	93.4	85.4	101.3	93.4	84.8	102	93.37	84.77	102
k_parent	-	0.0548	0.04	0.69	0.0548	0.039	0.071	0.05479	0.0411	0.07303
err [%]	-	13.46	-	-	13.5	-	-	13.46	-	-

Table S60: Results with confidence intervals for DFOP_lin_c evaluated with FOMC

Result	Input	KinGUII			CAKE			mkin		
		Estimate	From	To	Estimate	From	To	Estimate	From	To
parent_0	100	102.4	97.2	107.6	102.4	96.7	108	102	97	110
alpha	-	0.851	0.6	1.1	0.851	0.58	1.12	0.851	0.62	1.2
beta	-	6.46	2.87	10.06	6.46	2.56	10.37	6.46	3.5	12
err [%]	-	5.11	-	-	5.11	-	-	5.11	-	-

Table S61: Results with confidence intervals for DFOP_lin_c evaluated with DFOP

Result	Input	KinGUII			CAKE			mkin		
		Estimate	From	To	Estimate	From	To	Estimate	From	To
parent_0	100	103.8	99.2	108.4	103.8	98.7	108.8	103.8	98.72	108.8
k1	0.2	0.293	0.17	0.415	0.293	0.159	0.427	0.2928	0.1853	0.4625
k2	0.02	0.0225	0.017	0.028	0.0225	0.016	0.029	0.02245	0.01687	0.02989
g	0.5	0.47	0.376	0.566	0.47	0.367	0.574	0.4701	0.3692	0.5735
err [%]	-	2.97	-	-	2.97	-	-	2.966	-	-

Table S62: Results with confidence intervals for DFOP_lin_c evaluated with HS

Result	Input	KinGUII			CAKE			mkin		
		Estimate	From	To	Estimate	From	To	Estimate	From	To
parent_0	100	103.9	99.1	108.7	103.9	98.7	109.2	100.7	95.32	106.1
k1	-	0.135	0.103	0.166	0.135	0.1	0.169	0.09546	0.07736	0.1178
k2	-	0.0262	0.021	0.032	0.0262	0.02	0.032	0.02215	0.01569	0.03127
tb	-	4.6	3.21	5.99	4.6	3.08	6.12	8.406	5.81	12.16
err [%]	-	3.85	-	-	3.85	-	-	5.508	-	-

Table S63: Results for DFOP_lin_c evaluated with SFO_lin

Result	Input	DegKinM	KinGUII	OpenModel	mkin		
					Estimate	Lower	Upper
parent_0	100	103.7	103.7	103.7	103.7	100.8	106.6
k1	0.2	0.2881	0.289	0.2891	0.2327	0.1343	0.4031
k2	0.02	0.0222	0.0223	0.0223	0.02066	0.01479	0.02885
g	0.5	0.4735	0.4734	0.4734	0.3666	0.2249	0.536
k_m1	0.3	0.2323	0.2327	0.2334	0.9345	0.002344	1
k_m2	0.02	0.0206	0.02066	0.02064	0.289	0.2225	0.3754
f_parent_m1	0.5	0.3662	0.3666	0.3672	0.02225	0.01887	0.02625
f_m1_m2	0.7	0.9355	0.9345	1[a]	0.4734	0.4145	0.533
err parent [%]	-	2.97	2.97	-	2.968	-	-
err m1 [%]	-	15.2	10.8	-	10.8	-	-
err m2 [%]	-	4.92	3.3	-	3.307	-	-
err all [%]	-	-	4.48	-	4.479	-	-

[a] This parameter was constrained to the interval from 0 to 1 to obtain this result

SFO_par_a

Table S64: Synthetic dataset SFO_par_a

Time	parent	M1	M2
0	101.5		
0	101.2		
1	86.1	16.6	
1	79.7	17.6	4.1
3	53	30.1	8.8
3	50.2	35.5	7.2
7	25	56.8	14.7
7	24.2	59.5	15.8
14	1.5	71.7	19.8
14	9.6	67.2	14.6
28		59.5	11.9
28	3.5	61.9	10.3
60		46.7	6.9
60		40.8	5.7
90	0.6	31.4	4.1
90		37.4	4.3
120		26.8	6.3
120	3.5	25.9	

Table S65: Results for SFO_par_a evaluated with SFO_par

Result	Input	DegKinM	KinGUII	OpenModel	mkin		
					Estimate	Lower	Upper
parent_0	100	101.2	101.2	101.2	101.2	98.09	104.3
k_parent	0.2	0.2099	0.2103	0.2103	0.2104	0.1954	0.2265
k_m1	0.01	0.0098	0.00979	0.00979	0.009792	0.008763	0.01094
k_m2	0.02	0.0191	0.0191	0.0192	0.01914	0.01264	0.02899
f_parent_m1	0.8	0.7706	0.7709	0.7709	0.7709	-	-
f_parent_m2	0.2	0.1998	0.1999	0.1999	0.1999	-	-
err parent [%]	-	3.77	4.57	-	4.574	-	-
err m1 [%]	-	3.47	3.45	-	3.449	-	-
err m2 [%]	-	14.55	15.45	-	15.45	-	-
err all [%]	-	-	5.91	-	5.907	-	-

SFO_par_b

Table S66: Synthetic dataset SFO_par_b

Time	parent	M1	M2
0	103.5		
0	102.8		
1	91.8	19.5	
1	76.8	21.9	4.9
3	50.6	22.8	9
3	43.9	35.6	5.2
7	25.6	55.5	15.8
7	23.6	61.8	18.4
14		76.6	25.5
14	14.2	66.1	13.6
28		54.4	11
28	7.7	59.9	7.3
60		47.4	7.1
60		33.6	4.3
90	1.3	27.5	4.7
90		41.7	5.1
120		28.8	11.9
120	8.2	26.6	

Table S67: Results for SFO_par_b evaluated with SFO_par

Result	Input	DegKinM	KinGUII	OpenModel	mkin		
					Estimate	Lower	Upper
parent_0	100	102.5	102.5	102.5	102.5	95.26	109.8
k_parent	0.2	0.2193	0.2197	0.2198	0.2197	0.1849	0.2611
k_m1	0.01	0.0096	0.00965	0.00965	0.009645	0.007428	0.01252
k_m2	0.02	0.0181	0.0181	0.0181	0.0181	0.007176	0.04565
f_parent_m1	0.8	0.7414	0.7417	0.7416	0.7417	-	-
f_parent_m2	0.2	0.2017	0.2015	0.2015	0.2015	-	-
err parent [%]	-	9.89	12.48	-	12.48	-	-
err m1 [%]	-	8.16	8.15	-	8.147	-	-
err m2 [%]	-	31.46	32.2	-	32.2	-	-
err all [%]	-	-	14.62	-	14.62	-	-

SFO_par_c

Table S68: Synthetic dataset SFO_par_c

Time	parent	M1	M2
0	103.5		
0	102.8		
1	90	15.2	2.3
1	77.7	15.6	3.7
3	52.5	30.9	8.8
3	48.8	35.5	8.3
7	24.9	56.4	14.1
7	24.4	60.1	14.5
14	5.1	73.9	17.2
14	6.8	66.7	15.1
28		57.6	12.4
28	0.9	61.2	11.8
60		46.8	6.7
60		40.3	6.5
90		31.9	3.8
90		36.9	3.8
120		26.3	2.7
120	0.6	25.7	1.6

Table S69: Results for SFO_par_c evaluated with SFO_par

Result	Input	DegKinM	KinGUII	OpenModel	mkin		
					Estimate	Lower	Upper
parent_0	100	102.6	102.6	102.6	102.6	99.54	105.7
k_parent	0.2	0.2137	0.2141	0.2141	0.2141	0.1992	0.2301
k_m1	0.01	0.0098	0.00981	0.0098	0.00981	0.008793	0.01094
k_m2	0.02	0.0196	0.0197	0.0197	0.01967	0.0132	0.0293
f_parent_m1	0.8	0.7559	0.7562	0.7563	0.7562	-	-
f_parent_m2	0.2	0.1927	0.1929	0.1929	0.1929	-	-
err parent [%]	-	2.76	3.2	-	3.202	-	-
err m1 [%]	-	3.66	3.64	-	3.642	-	-
err m2 [%]	-	4.34	4.26	-	4.258	-	-
err all [%]	-	-	4.52	-	4.515	-	-

DFOP_par_a

Table S70: Synthetic dataset DFOP_par_a

Time	parent	M1	M2
0	101.5		
0	101.2		
1	85.5	13.2	0.6
1	79.1	14.2	8
3	54.9	18.8	17.1
3	52	24.3	15.5
7	35	31.8	25.8
7	34.2	34.5	26.9
14	19.2	33.9	31.8
14	27.2	29.4	26.7
28	14	16.4	25.6
28	20.3	18.7	24
60	5.8	8.7	22
60	6.2	2.8	20.8
90	5.5	0.9	18
90	1	7	18.2
120		3.4	18
120	6.2	2.4	11.4

Table S71: Results for DFOP_par_a evaluated with DFOP_par

Result	Input	DegKinM	KinGUII	OpenModel	mkin		
					Estimate	Lower	Upper
parent_0	100	101	101	101	101	97.32	104.7
k1	0.3	0.3043	0.3049	0.3049	0.3049	0.2521	0.3687
k2	0.02	0.0214	0.0214	0.0214	0.02137	0.01443	0.03166
g	0.7	0.7117	0.7121	0.7121	0.7121	0.637	0.7771
k_m1	0.04	0.0438	0.0439	0.0439	0.04387	0.03445	0.05587
k_m2	0.01	0.0095	0.00948	0.00948	0.009482	0.007201	0.01248
f_parent_m1	0.6	0.5916	0.592	0.592	0.592	-	-
f_parent_m2	0.4	0.3869	0.3867	0.3867	0.3867	-	-
err parent [%]	-	4.43	4.64	-	4.639	-	-
err m1 [%]	-	9.12	8.87	-	8.873	-	-
err m2 [%]	-	6.41	6.41	-	6.407	-	-
err all [%]	-	-	6.83	-	6.826	-	-

DFOP_par_b

Table S72: Synthetic dataset DFOP_par_b

Time	parent	M1	M2
0	103.5		
0	102.8		
1	91.2	16.1	
1	76.2	18.5	8.7
3	52.4	11.6	17.3
3	45.8	24.3	13.5
7	35.5	30.4	26.9
7	33.6	36.8	29.5
14	13.1	38.8	37.6
14	31.9	28.2	25.6
28	9.7	11.3	24.7
28	24.5	16.8	21
60	1.4	9.4	22.3
60	2.4		19.4
90	6.3		18.6
90		11.3	19
120		5.3	23.7
120	10.9	3.2	8.3

Table S73: Results for DFOP_par_b evaluated with DFOP_par

Result	Input	DegKinM	KinGUII	OpenModel	mkin		
					Estimate	Lower	Upper
parent_0	100	103.6	103.6	103.6	103.6	95.49	111.6
k1	0.3	0.3433	0.3411	0.3411	0.3412	0.2306	0.5047
k2	0.02	0.0208	0.0205	0.0206	0.02054	0.00834	0.05061
g	0.7	0.7234	0.726	0.7259	0.726	0.5689	0.8418
k_m1	0.04	0.0354	0.0357	0.0357	0.03566	0.01997	0.06366
k_m2	0.01	0.0091	0.0091	0.0091	0.009086	0.005033	0.0164
f_parent_m1	0.6	0.519	0.5207	0.5211	0.5207	-	-
f_parent_m2	0.4	0.3767	0.3766	0.3767	0.3766	-	-
err parent [%]	-	9.31	10.21	-	10.21	-	-
err m1 [%]	-	20.89	22.11	-	22.11	-	-
err m2 [%]	-	9.23	9.54	-	9.541	-	-
err all [%]	-	-	14.92	-	14.92	-	-

DFOP_par_c

Table S74: Synthetic dataset DFOP_par_c

Time	parent	M1	M2
0	103.5		
0	102.8		
1	89.3	11.7	5.8
1	77.1	12	7.6
3	54.2	21.1	17.1
3	50.5	24.3	16.4
7	35	32	25.5
7	34.3	34.1	26.1
14	21.1	32.9	30.3
14	25.7	29.6	26.9
28	15.8	18.3	25.9
28	18.5	19.5	24.9
60	8.2	8.3	21.9
60	8.3	6.8	21.3
90	5.1	3.3	17.8
90	4.2	4.4	17.8
120	2.1	2.1	15.3
120	3.3	2	12.9

Table S75: Results for DFOP_par_c evaluated with DFOP_par

Result	Input	DegKinM	KinGUII	OpenModel	mkin		
					Estimate	Lower	Upper
parent_0	100	103	103	103	103	100.4	105.6
k1	0.3	0.3261	0.3263	0.3263	0.3263	0.2879	0.3697
k2	0.02	0.0202	0.0202	0.0202	0.02024	0.01581	0.02591
g	0.7	0.7125	0.713	0.713	0.713	0.6679	0.7542
k_m1	0.04	0.0387	0.0389	0.0388	0.03886	0.03297	0.04579
k_m2	0.01	0.0095	0.0095	0.0095	0.009532	0.007878	0.01153
f_parent_m1	0.6	0.5558	0.5565	0.5564	0.5565	-	-
f_parent_m2	0.4	0.3783	0.3784	0.3784	0.3784	-	-
err parent [%]	-	3.05	3.05	-	3.051	-	-
err m1 [%]	-	5.41	5.07	-	5.067	-	-
err m2 [%]	-	3.19	3.17	-	3.173	-	-
err all [%]	-	-	4.03	-	4.033	-	-

Results for experimental test datasets

Test data UBA 2014 River

Table S76: Experimental test dataset UBA_2014_WS_river

Time	parent_w	parent_s
0	98	0
0	95.1	0.1
2	45.1	4.8
2	35.4	5.4
8	2.4	11.3
8	15.1	8.8
21	0.03	3.3
21	0.03	5.6
55	0.03	
55	0.03	1.1
105	0.03	
105	0.03	0.2

Table S77: Results for UBA_2014_WS_river

Result	KinGUII	OpenModel	mkin
parent_w_0	95.99	96	95.99
k_parent_w_sink	0.3604	0.3607	0.3604
k_parent_w_parent_s	0.0603	0.0603	0.06031
k_parent_s_sink	2.3e-14	1e-05	5.109e-11
k_parent_s_parent_w	0.0742	0.075	0.0742
err all [%]	9.54	-	9.25
err parent w [%]	6.38	-	6.38
err parent s [%]	22.7	-	20.88

Test data UBA 2014 Pond

Table S78: Experimental test dataset UBA_2014_WS_pond

Time	parent_w	parent_s
0	98	0
0	97.7	0
2	60.5	11.9
2	53.8	12.7
8	5.2	12.2
8	2.8	11.5
21	1.6	6.9
21		5.2
55		1
55		0.9
105		0.3
105		0.5

Table S79: Results for UBA_2014_WS_pond

Result	KinGUII	OpenModel	mkin
parent_w_0	99.2	99.2	99.2
k_parent_w_sink	0.227	0.2256	0.227
k_parent_w_parent_s	0.0779	0.0789	0.07785
k_parent_s_sink	0.0984	0.1012	0.09833
k_parent_s_parent_w	2.3e-14	1e-07	2.123e-11
err all [%]	10.3	-	10.31
err parent w [%]	7.63	-	7.63
err parent s [%]	17.9	-	17.88

Test data UBA 2014 Soil

Table S80: Experimental test dataset UBA_2014_soil

Time	parent	M1	M2	M3
0	78.3	0	0	0
1	67	1.8	2.5	0.6
2	59.6	2.5	2.7	0.7
6	34.9	2	6.2	3.5
16	14	0.6	6.3	7.1
34	3.8	0.8	3.5	8.4
64	2	0.6	2.1	8.3
100	1.5	0.6	1.9	5.4

Table S81: Results for UBA_2014_soil

Result	KinGUII	CAKE	OpenModel	mkin
parent_0	76.6	76.7	76.7	76.55
k_parent	0.121	0.121	0.122	0.1208
k_M1	0.843	0.844	0.864	0.8426
k_M2	0.042	0.042	0.045	0.04211
k_M3	0.011	0.011	0.012	0.01123
f_parent_to_M1	0.322	0.322	0.318	0.3224
f_parent_to_M2	0.161	0.161	0.163	0.161
f_M1_to_M3	0.279	0.278	0.276	0.2792
f_M2_to_M3	0.557	0.557	0.584	0.5564
err all [%]	9.65	9.65	-	9.65
err parent [%]	4.72	4.72	-	4.72
err M1 [%]	26.55	26.6	-	26.55
err M2 [%]	20.33	20.3	-	20.33
err M3 [%]	5.2	5.2	-	5.2

References

FOCUS (2006). Guidance document on estimating persistence and degradation kinetics from environmental fate studies on pesticides in EU registration. Report of the FOCUS Work Group on Degradation Kinetics, EC Doc. Ref. Sanco/10058/2005, version 2.0, Work Group on Degradation Kinetics of FOCUS (FOrum for the Co-ordination of pesticide fate models and their USe)

FOCUS (2014). Generic guidance for estimating persistence and degradation kinetics from environmental fate studies on pesticides in EU registration. Report Version 1.1

Ranke, J. (2011). kinfit — routines for fitting kinetic models to chemical degradation data. R package vignette. URL https://cgit.jrwb.de/kinfit/plain/inst/doc/kinfit.pdf?id=3a6b9f52c74d6ef88a8d32c50e42864b3f251719

Ranke, J., Wöltjen, J. and Meinecke, S. (2018). Comparison of software tools for kinetic evaluation of chemical degradation data. *Submitted to Environmental Sciences Europe*
